# The potential for non-adaptive origins of evolutionary innovations in central carbon metabolism

**DOI:** 10.1186/s12918-016-0343-7

**Published:** 2016-10-21

**Authors:** Sayed-Rzgar Hosseini, Andreas Wagner

**Affiliations:** 1 Institute of Evolutionary Biology and Environmental Studies, University of Zurich, Bldg. Y27, Winterthurerstrasse 190, CH-8057 Zurich, Switzerland; 2 The Swiss Institute of Bioinformatics, Bioinformatics, Quartier Sorge, Batiment Genopode, 1015 Lausanne, Switzerland; 3 The Santa Fe Institute, 1399 Hyde Park Road, Santa Fe, NM 87501 USA

**Keywords:** Exaptation, Innovation, Central carbon metabolism, Exhaustive genotype-phenotype mapping

## Abstract

**Background:**

Biological systems are rife with examples of pre-adaptations or exaptations. They range from the molecular scale – lens crystallins, which originated from metabolic enzymes – to the macroscopic scale, such as feathers used in flying, which originally served thermal insulation or waterproofing. An important class of exaptations are novel and useful traits with non-adaptive origins. Whether such origins could be frequent cannot be answered with individual examples, because it is a question about a biological system’s *potential* for exaptation.

We here take a step towards answering this question by analyzing central carbon metabolism, and novel traits that allow an organism to survive on novel sources of carbon and energy. We have previously applied flux balance analysis to this system and predicted the viability of 10^15^ metabolic genotypes on each of ten different carbon sources.

**Results:**

We here use this exhaustive genotype-phenotype map to ask whether a central carbon metabolism that is viable on a given, focal carbon source *C* – the equivalent of an adaptation in our framework – is usually or rarely viable on one or more other carbon sources *C*_*new*_ – a potential exaptation. We show that most metabolic genotypes harbor potential exaptations, that is, they are viable on one or more carbon sources *C*_*new*_. The nature and number of these carbon sources depends on the focal carbon source *C* itself, and on the biochemical similarity between *C* and *C*_*new*_. Moreover, metabolisms that show a higher biomass yield on *C*, and that are more complex, i.e., they harbor more metabolic reactions, are viable on a greater number of carbon sources *C*_*new*_.

**Conclusions:**

A high potential for exaptation results from correlations between the phenotypes of different genotypes, and such correlations are frequent in central carbon metabolism. If they are similarly abundant in other metabolic or biological systems, innovations may frequently have non-adaptive (“exaptive”) origins.

**Electronic supplementary material:**

The online version of this article (doi:10.1186/s12918-016-0343-7) contains supplementary material, which is available to authorized users.

## Background

One of the most fundamental questions in evolutionary biology regards the origin of qualitatively new and beneficial traits, i.e., evolutionary innovations [1]. On the one hand, such traits can originate as adaptations that help an organism survive or reproduce. On the other hand, they can also have non-adaptive origins as pre-adaptations or exaptations [2, 3]. Darwin was the first to pay attention to the importance of pre-adaptation when he said that “an organ originally constructed for one purpose… may be converted to one for a widely different purpose“ [4]. Later on, multiple lines of evidence from the organismal to the molecular scale confirmed the importance of exaptations as important sources of evolutionary innovation [5–7]. A textbook example involves feathers, which are made of keratins, the same proteins that constitute the scales of reptiles. Feathers most likely originally served for thermoregulation and waterproofing, and were only later “exapted” for flying [2]. Many crystallins, light-refracting proteins in eye lenses, originated as metabolic enzymes [8]. More generally, many genes have been coopted into various developmental and physiological functions by changing their patterns of regulation [5]. For example, the Hedgehog signaling protein, responsible for proper limb development in mammals, has also been coopted to paint eyespots in butterflies, and it helps shape feathers in birds [7]. Exaptations may also have been important in human evolution [9].

It is easy to find examples of exaptations, but much more difficult to find out how frequently any one biological system can bring forth non-adaptive traits that could turn into exaptations. This is not a question about natural history, but about a biological system’s *potential* for exaptation. It is the focus of this contribution. The question can only be answered in systems where one can study, either experimentally or computationally, many genotypes and the phenotypes that they form [10–14]. In doing so, one can ask whether a beneficial phenotypic trait frequently entails other traits with the potential to become an exaptation.

Metabolism is a well-suited system for this purpose, and for two main reasons. First, metabolism is a source of multiple evolutionary innovations, especially in microorganisms. For example, microorganisms have acquired the ability to extract energy from non-natural substances, including toxic compounds [15–18]. By producing novel molecules such as ectoine or glycine betaine, halophilic bacteria can tolerate high salt concentrations [19]. And microbial isolates from pristine soils show not only resistance to a wide range of antibiotics, but many of them are also capable of using these molecules as sources of energy and chemical elements [20].

Second, one can predict novel metabolic phenotypes using computational tools such as flux balance analysis (FBA) for multiple metabolic genotypes [21–26]. The metabolic genotype of an organism is a string of DNA encoding the enzymes catalyzing metabolic reactions, but for computational expediency, a more compact representation of a metabolic genotype based on reactions rather than genes is often used [21–24, 27, 28]. Specifically, given a known “universe” of enzyme-catalyzed biochemical reactions, one can represent the metabolic genotype of an organism as a binary vector whose *i*-th entry corresponds to the *i*-th reaction in this reaction universe [29]. If an organism’s genome encodes an enzyme capable of catalyzing a given reaction, the corresponding entry in the genotype vector will be one and zero otherwise. The collection of all such vectors constitutes a metabolic genotype space, and any one organism’s metabolic genotype can be thought of as a point in this space. FBA can predict metabolic phenotypes, such as viability (the ability of a metabolism to sustain life in a given spectrum of chemical environments) for any one metabolic genotype. Importantly, FBA-based predictions of viability are in good agreement with experimental data [27, 30–35].

In previous work, we analyzed potential exaptations in genome-scale metabolisms [36]. This work relied on sampling of metabolic genotypes from a vast metabolic genotype space [14, 26]. Because any such sample represents a tiny fraction of the whole space, we here complement this analysis with a more comprehensive approach that examines all members of a genotype space. This is impossible for genome-scale metabolisms, because of their astronomical numbers, but it is possible for smaller-scale metabolic systems, such as a genotype space defined by the 51 biochemical reactions of central carbon metabolism [29].

Central carbon metabolism is a small but crucial part of metabolism, because it plays a pivotal role in life by extracting energy from extracellular carbon sources (Additional files 1 and 2). It includes the interrelated biochemical pathways of glycolysis, gluconeogenesis, the pentose-phosphate pathway (PP), and the tricarboxylic acid cycle (TCA), which are supplemented by anaplerotic reactions and the glyoxylate shunt [37]. Glycolysis creates high-energy compounds like ATP and NADH and converts glucose into pyruvate. The tricarboxylic acid cycle (TCA) generates ATP, NADH, and amino acid precursors from acetyl-CoA, which results from oxidation of the glycolytic end product pyruvate. The pentose-phosphate pathway produces NADPH and pentose sugars for anabolic reactions. Finally, the reactions of the oxidative phosphorylation pathway participate in production of ATP from NADH.

We here use the genotype-phenotype map of central carbon metabolism to ask how often metabolisms viable on a given carbon source *C* can survive on one or more other carbon sources *C*_*new*_. We show that this is the case for most metabolisms, and we analyze which properties of a metabolism facilitate its potential for exaptation. These properties include the complexity of a metabolism and its efficiency in converting nutrients into biomass. We emphasize that we are not focused on the evolutionary history of central carbon metabolism, but on the potential for its biochemical pathways to bring forth exaptations.

## Results

### The genotype space of central carbon metabolism

The genotype space we consider includes all 2^51^≈10^15^ metabolic genotypes whose reactions form a subset of the 51 internal reactions of the central carbon metabolism of *E.coli* [29]. Each genotype specifies a chemical reaction network that we refer to as a central carbon metabolism. We call a genotype (metabolism) viable on a given carbon source, if it can synthesize each one of 13 biomass precursors from this source in an otherwise minimal chemical environment (Additional files 1 and 2) [38]. In previous work, we determined the viability of each of the 2^51^ genotypes on ten different carbon sources [39–41], and found that only a tiny fraction of genotypes can sustain life on any one carbon source. This fraction ranges from 10^−8^(on acetate) to 10^−6^ (on glucose), corresponding to between ≈10^7^ and ≈10^9^ genotypes that are viable on acetate and glucose, respectively. Genotypes viable on a given carbon source form a connected network in genotype space, which implies that different metabolisms can be converted into each other in few viability-preserving mutational steps [39]. We use *E.coli* central metabolism as a departure point for our analysis for two reasons. First, it is small enough to be amenable to exhaustive genotype-phenotype mapping, yet large enough to show multiple different phenotypes. Second, *E.coli* central carbon metabolism is especially well characterized and reasonably complete [38, 42]. Other genotypes in the genotype space we examine correspond to incomplete variants, such as those lacking a complete citric acid cycle or having incomplete pentose-phosphate pathway.

The fundamental question we ask here is whether a metabolism that is viable on a specific focal carbon source *C* is usually also viable on one or more other carbon sources *C*_*new*_, which corresponds to a potential exaptation or preadaptation in the framework of our computational analysis. In a previous analysis, we had asked this question for randomly sampled genome-scale metabolisms required to be viable on a specific carbon source *C* [36], and we here extend this approach to all ≈10^15^ central carbon metabolisms whose phenotypes we have previously exhaustively enumerated [39–41].

### High potential for exaptation in central carbon metabolism

**Fig. 1:**
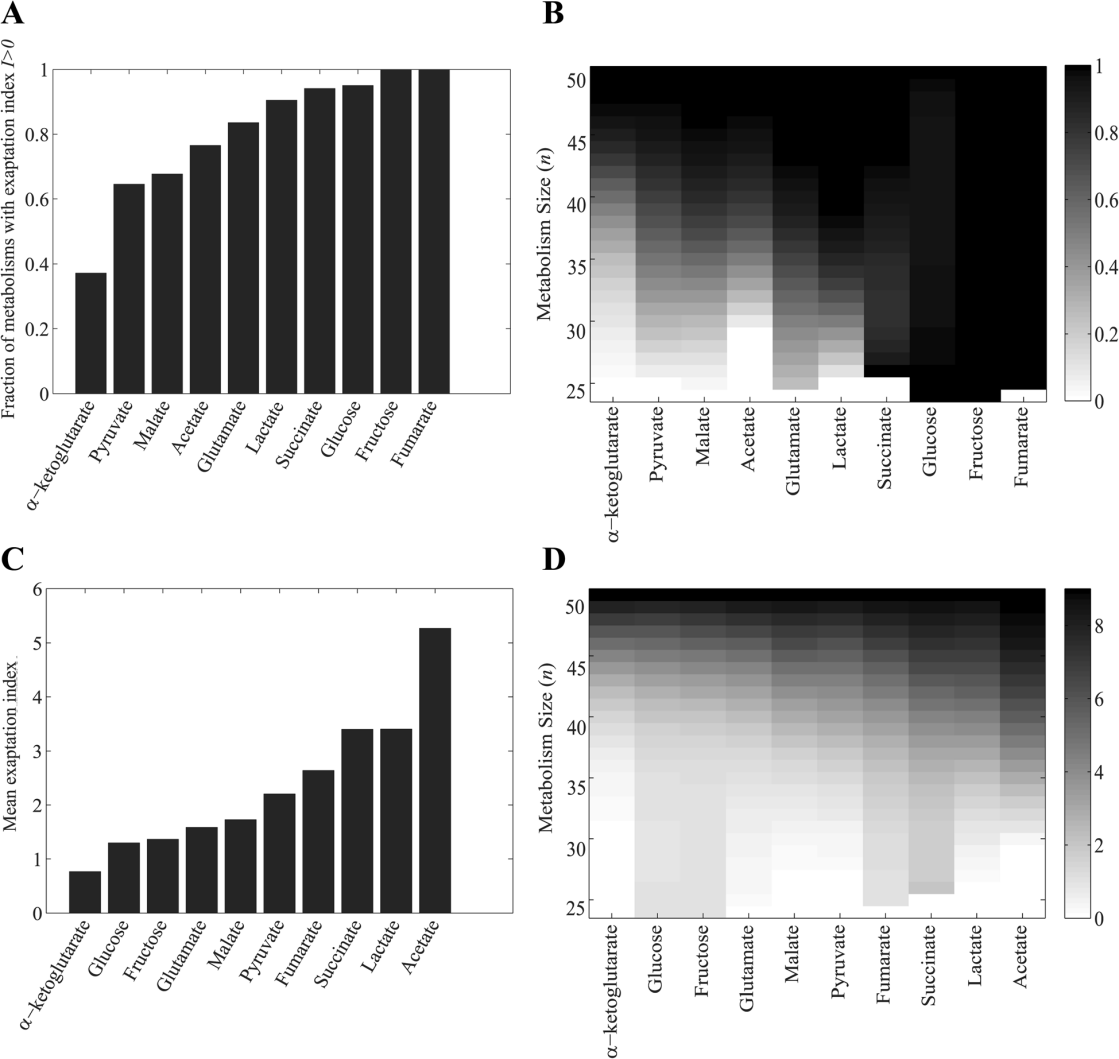
High exaptation potential in central carbon metabolisms. **a** Fraction of metabolisms with exaptation index *I>0* (*y*-axis) viable on some carbon source *C* (*x*-axis); **b** fraction of metabolisms (coded by shade of *grey*, see legend) with exaptation index *I>0* that are viable on some carbon source *C* (*x*-axis) and have a given size (*y*-axis), **c** mean exaptation index of metabolisms viable on some carbon source *C* (*x*-axis), and **d** mean exaptation index (coded by shade of *grey*, see legend) of metabolisms viable on some carbon source *C* (*x*-axis) and with a given size (*y*-axis). *White* in (**b**) and (**d**) corresponds to metabolisms whose size is too small for viability on *C*

We first defined an exaptation index *I* as the number of carbon sources *C*_*new*_ on which a metabolism is viable (in addition to the carbon source *C*) [36]. We then asked what fraction of metabolisms viable on *C* have *I > 0*, i.e., they are exapted to at least one additional carbon source. Figure 1a shows that for all ten focal carbon sources *C* we consider here except one, the majority of metabolisms are viable on at least one carbon source *C*_*new*_. For example, 95 % of metabolisms viable on glucose are also viable on at least one additional carbon source. The one exception is α-ketoglutarate for which only 38 % of viable metabolisms are also viable on additional carbon sources (Fig. 1a). Another extreme is represented by metabolisms viable on fructose and fumarate, all of which are viable on additional carbon sources. The reason is that all metabolisms viable on fructose are also viable on glucose, and all metabolisms viable on fumarate are also viable on succinate. In sum, central carbon metabolism harbors great potential for exaptation.

One can partition metabolic genotype space according to the complexity or size of genotypes, defined as the number of reactions *n* in a metabolism [40]. Any one metabolism needs to have a minimal size *n* for viability, which depends on the carbon source considered, and ranges from *n = 23* for glucose to *n = 30* for acetate. We next asked if the fraction of metabolisms with *I >0* depends on this metabolism size. Figure 1b shows that it does. For any one carbon source *C*, more complex metabolisms have a higher potential for exaptation. The exceptions are metabolisms viable on fumarate and fructose, because all of them have *I > 0*, regardless of size.

We next determined the average exaptation index among all viable metabolisms, which indicates the average number of additional carbon sources *C*_*new*_ that a metabolism viable on some carbon source *C* is also viable on. This average exaptation index exceeds 1 in all of the carbon sources except for α-ketoglutarate, where it is 0.88 (Fig. 1c). For acetate, this index has the largest value (*I = 5.27*), which implies that a metabolism viable on acetate will, on average, be viable on more than five of the remaining nine carbon sources. The index also increases with increasing metabolism size (Fig. 1d), meaning that larger metabolisms are viable on more carbon sources *C*_*new*_. Furthermore, we observed that the exaptation index varies widely among metabolisms of the same size and viable on the same carbon source (Additional file 3).

In a final analysis, we asked whether high exaptation potential might be caused preferentially by reactions that are disconnected from the rest of metabolism. Such disconnected reactions must fulfill at least one of the following two criteria. First, their products are neither biomass precursors nor substrates of any other reaction of a given metabolism. Second, at least one of their substrates is neither a product of other reactions nor a nutrient taken up from the environment. To find out how disconnected reactions affect exaptation potential, we eliminated from our analysis those metabolisms harboring such reactions, and observed that the exaptation indices remain almost unchanged (Additional file 4). The incidence of disconnected reactions does not strongly affect the exaptation potential of central carbon metabolism.

In sum, viability on a given carbon source generally entails viability on other carbon sources, and often on multiple such carbon sources. Thus, central carbon metabolism has a high potential for exaptation. This potential increases with metabolic complexity, i.e., with the number of reactions in a metabolism.

### Minimal central carbon metabolisms also harbor exaptation potential

A special role in our analysis is played by metabolisms that are *minimal*. We define them as metabolisms from which not a single reaction can be removed without abolishing viability on the focal carbon source. We note that there may be multiple such metabolisms, and that they are not necessarily the smallest possible metabolisms viable on this carbon sources. They are important, because they harbor only essential reactions. If exaptation potential depends on non-essential reactions, then it is possible that such minimal metabolisms harbor no exaptation potential.

To find out, we first identified all minimal central carbon metabolisms viable on a given focal carbon source (Table 1). For example, 161 minimal central carbon metabolisms are viable on glucose, and their size varies from 23 to 30 reactions [39].

**Table 1:** Exaptation potential of minimal metabolisms

Focal carbon source	Number of minimal metabolisms	Size range	Number (percentage) of minimal metabolisms with *I>0*
Fructose	146	[23–30]	146 (100.00 %)
Fumarate	456	[26–32]	456 (100.00 %)
Glucose	161	[23–30]	154 (95.65 %)
Succinate	348	[27–32]	304 (87.36 %)
Lactate	180	[26–34]	144 (80.00 %)
Malate	456	[25–32]	176 (38.60 %)
Glutamate	187	[26–32]	72 (38.50 %)
Acetate	76	[30–36]	16 (21.05 %)
Pyruvate	569	[26–34]	96 (16.87 %)
α-ketoglutarate	970	[25–32]	80 (8.25 %)

Columns, from left to right, indicate the focal carbon source, the number of minimal metabolisms that are viable on this carbon source, the size range of these metabolisms, and the number (percentage) of minimal metabolisms with exaptation index *I>0*

The ten focal carbon sources in Table 1 can be subdivided into two groups. In the first group (fructose, fumarate, glucose, succinate and lactate), the vast majority (80–100 %) of minimal central carbon metabolisms have exaptation indices *I > 0*. For example, among the 161 minimal metabolisms viable on glucose, 154 (95.7 %) can survive on at least one additional carbon source (146 on one, and eight on two additional carbon sources). Three of these 154 minimal metabolisms have the smallest possible size for metabolisms viable on glucose (*n = 23* reactions), and each of these three is viable on one additional carbon source. For the second group of focal carbon sources (Table 1, malate, glutamate, acetate, pyruvate, and α-ketoglutarate), fewer than 50 % of minimal metabolisms show an exaptation index *I* > 0. For example, among the 76 minimal metabolisms viable on acetate, 60 are only viable on acetate and only 16 (21 %) of them can survive on another carbon source. In sum, there are clear differences among carbon sources in the exaptation potential of minimal metabolisms. However, on all carbon sources some minimal metabolisms show exaptation potential, and on half of the carbon sources the vast majority of minimal metabolisms does. Non-essential reactions are not solely responsible for the exaptation potential of central carbon metabolisms.

**Fig. 2:**
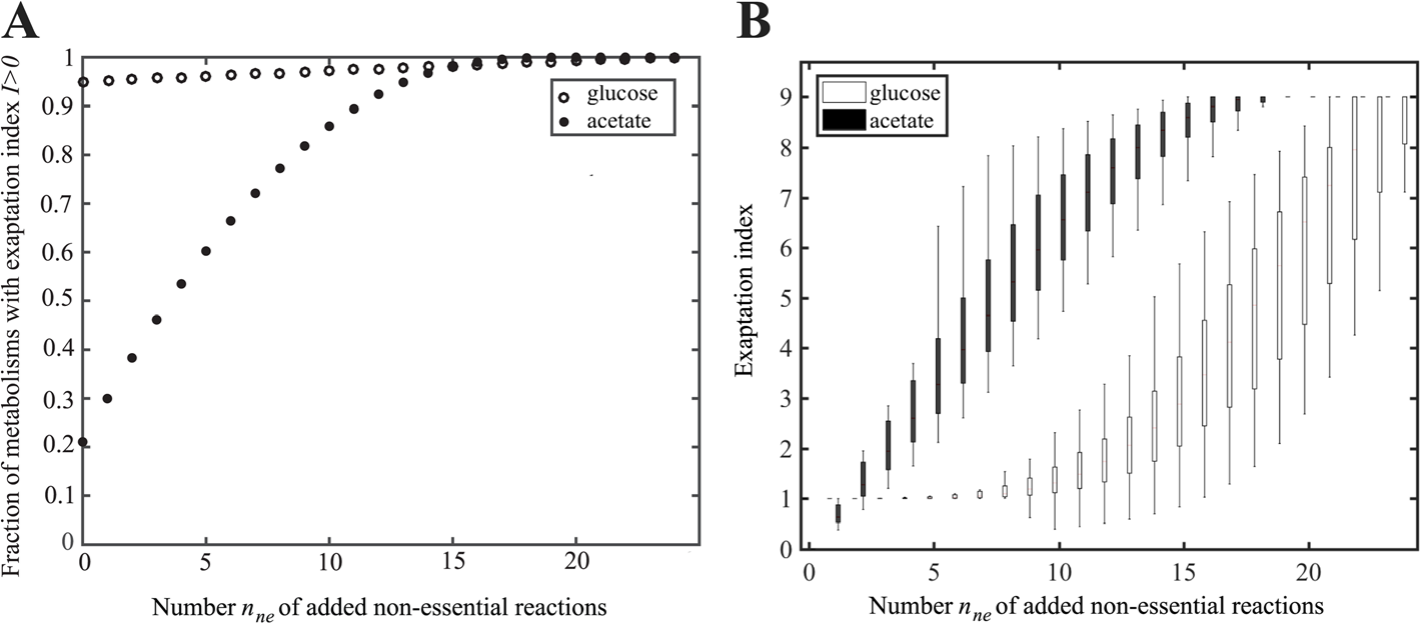
Exaptation potential and non-essential reactions. Vertical axes show (**a**) the fraction of metabolisms with exaptation index *I>0*, and (**b**) the exaptation index itself, among metabolisms generated by adding a given number *n*_ne_ of non-essential reactions (*x*-axis) to the minimal metabolisms viable on glucose (open circles/boxes), and acetate (filled circles/boxes). Boxes span the 25-th to 75-th percentile, and whiskers indicate maxima and minima. Note that this analysis is exhaustive, meaning that (*i*) all minimal metabolisms viable on glucose (161), and acetate (76) are considered, and (*ii*) all possible *n*_ne_ -tuples of non-essential reactions (*x*-axis) have been added to each minimal metabolism

That being said, reactions that are non-essential on any one carbon source do play a role in increasing a metabolism’s exaptation potential, but the importance of this role depends on the carbon source. We demonstrated this with the following approach, applied to all carbon sources, all minimal metabolisms for each carbon source, and all possible numbers *n*_ne_ of non-essential reactions. We identified all *n*_ne_ -tuples of such reactions, i.e., reactions that are not already part of the metabolism, added each *n*_ne_-tuple to the minimal metabolism, and determined the exaptation index *I* of the resulting metabolism. Figure 2a shows the number of added non-essential reactions together with the fraction of metabolisms with an exaptation index *I* >0, for two representative carbon sources from the two groups, glucose (group 1) and acetate (group 2). For glucose, where most minimal metabolisms already have exaptation potential, adding non-essential reactions cannot strongly increase this potential. Specifically, the fraction of metabolisms with exaptation index (*I > 0*) grows very slowly and it does not reach one even after addition of 20 non-essential reactions to some of the minimal metabolisms. Figure 2b shows the exaptation index itself as a function of the number *n*_ne_ of added reactions. For glucose, it remains nearly unchanged after adding five non-essential reactions to minimal networks, and starts to increase only thereafter. In contrast, for acetate, where the fraction of metabolisms with *I > 0* is low for *n*_*ne*_ *= 0*, this fraction rises rapidly, to over 60 % after adding five reactions, and to 100 % after adding 17 reactions (Fig. 2a). Moreover, the exaptation index itself increases rapidly. It surpasses the exaptation index of minimal metabolisms viable on glucose after adding merely three non-essential reactions, and increases rapidly thereafter as well. In sum, even minimal metabolisms have some exaptation potential, and in those with low exaptation potential, the addition of non-essential reactions increases this potential to the greatest extent.

### Metabolisms viable on a given focal carbon source can be pre-adapted to a wide variety of other carbon sources

Our analysis thus far has not addressed the question whether *different* metabolisms viable on some carbon source *C* show exaptation to *different* carbon sources *C*_*new*_. To answer this question, we separated metabolisms according to their focal carbon source *C* and their numbers of reactions, and determined the number of carbon sources *C*_*new*_ on which metabolisms in each of these categories are viable. For all except the smallest metabolisms (*n < 30*), at least one metabolism in each category is viable on each of the nine possible carbon sources *C*_*new*_ (Additional file 5). In other words, regardless of the focal carbon source *C*, exaptation is possible on every single alternative carbon source.

For the next step of our analysis, we represented viability on each of the nine carbon sources *C*_*new*_ as a binary phenotype vector. For any one metabolism, this vector contains a one for each carbon source *C*_*new*_, on which that metabolism is viable, and a zero otherwise. We defined the phenotypic distance between a given pair of metabolisms as the Hamming distance between these phenotype vectors. The greater this distance is for two metabolisms, the larger is the number of carbon sources *C*_*new*_ on which one metabolism is viable and the other is not.

**Fig. 3:**
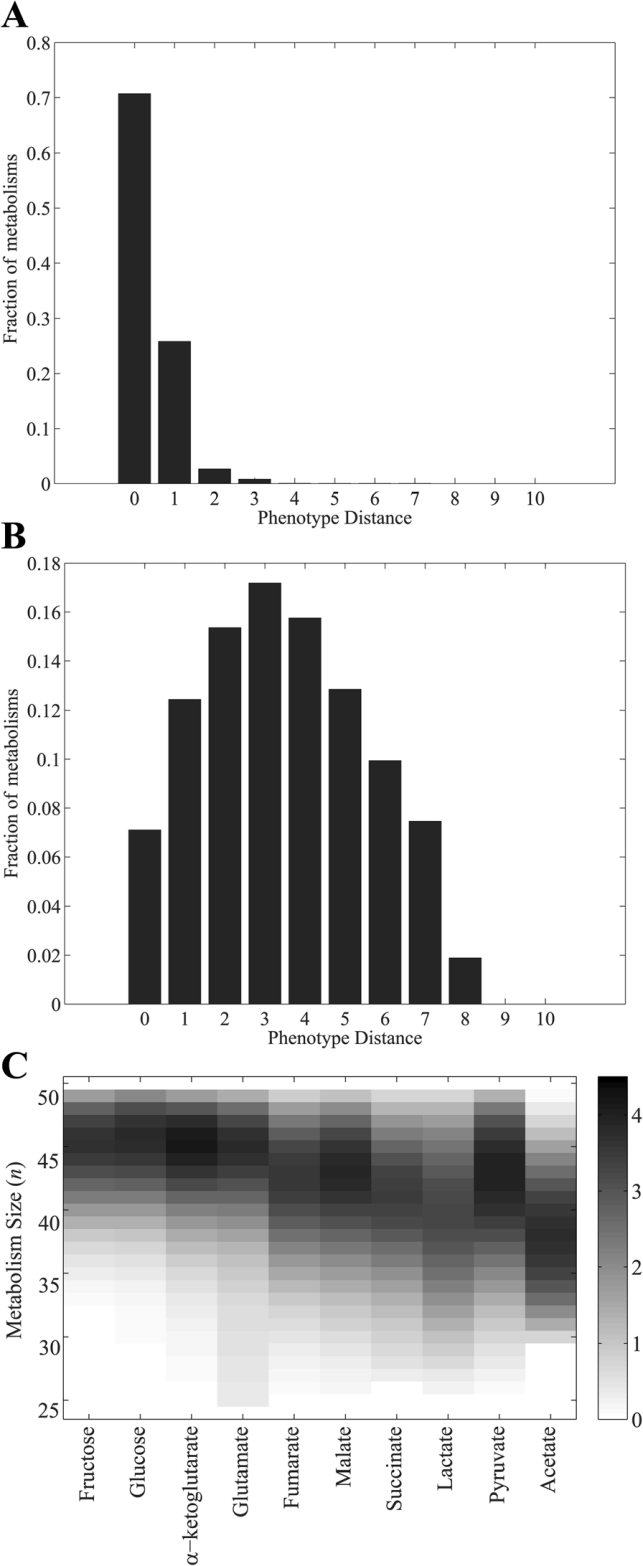
Metabolisms can preadapt to a wide variety of carbon sources. Panels **a** and **b** show histogram of the phenotype distance (*x*-axis), for metabolisms of size (**a**) 30, and (**b**) 45 viable on glucose as carbon source *C*. **c** Mean phenotypic distance (coded by shade of *grey*, see legend) of metabolisms viable on a focal carbon source (*x*-axis) and with a given number of reactions *n* (*y*-axis)

Figures 3a and b show examples of the distribution of the phenotype distance for metabolisms of *n = 30* and *n = 45* reactions viable on the focal carbon source glucose. 29.28 % (*n = 30*) and 92.89 % (*n = 45*) of all metabolism pairs have phenotypic distance larger than or equal to one, and 32.15 % (*n = 45*) of all metabolism pairs have phenotypic distance larger than or equal to five. Phenotypic distances can reach values up to eight, meaning that two metabolisms may share viability on only one of the nine possible carbon sources *C*_*new*_. (See also Additional file 6 for the remaining glucose panels and Additional file 7, where *C* is pyruvate). Figure 3c shows the mean phenotypic distances for metabolisms of different sizes *n* and focal carbon sources *C*. It illustrates that large phenotypic distances are not peculiarities of metabolism pairs viable on glucose or pyruvate. Also, for each focal carbon source *C*, phenotypic distance generally increases with metabolism size. The only exception involves the largest metabolisms (*n > 48*), where the average phenotypic distance is low and decreases with increasing *n*. The reason is that the largest metabolisms are highly likely to be viable on all ten carbon sources, which lowers their phenotypic distance. Similar observations hold for metabolisms without disconnected reactions (Additional files 8, 9 and 10).

In sum, different metabolisms viable on a given carbon source are usually exapted to different additional carbon sources, and this exaptive diversity increases with a metabolism’s size. The exaptation potential of central carbon metabolism can give rise to multiple different metabolic innovations.

### The potential for pre-adaptation depends on the biochemical similarity between carbon sources

**Fig. 4:**
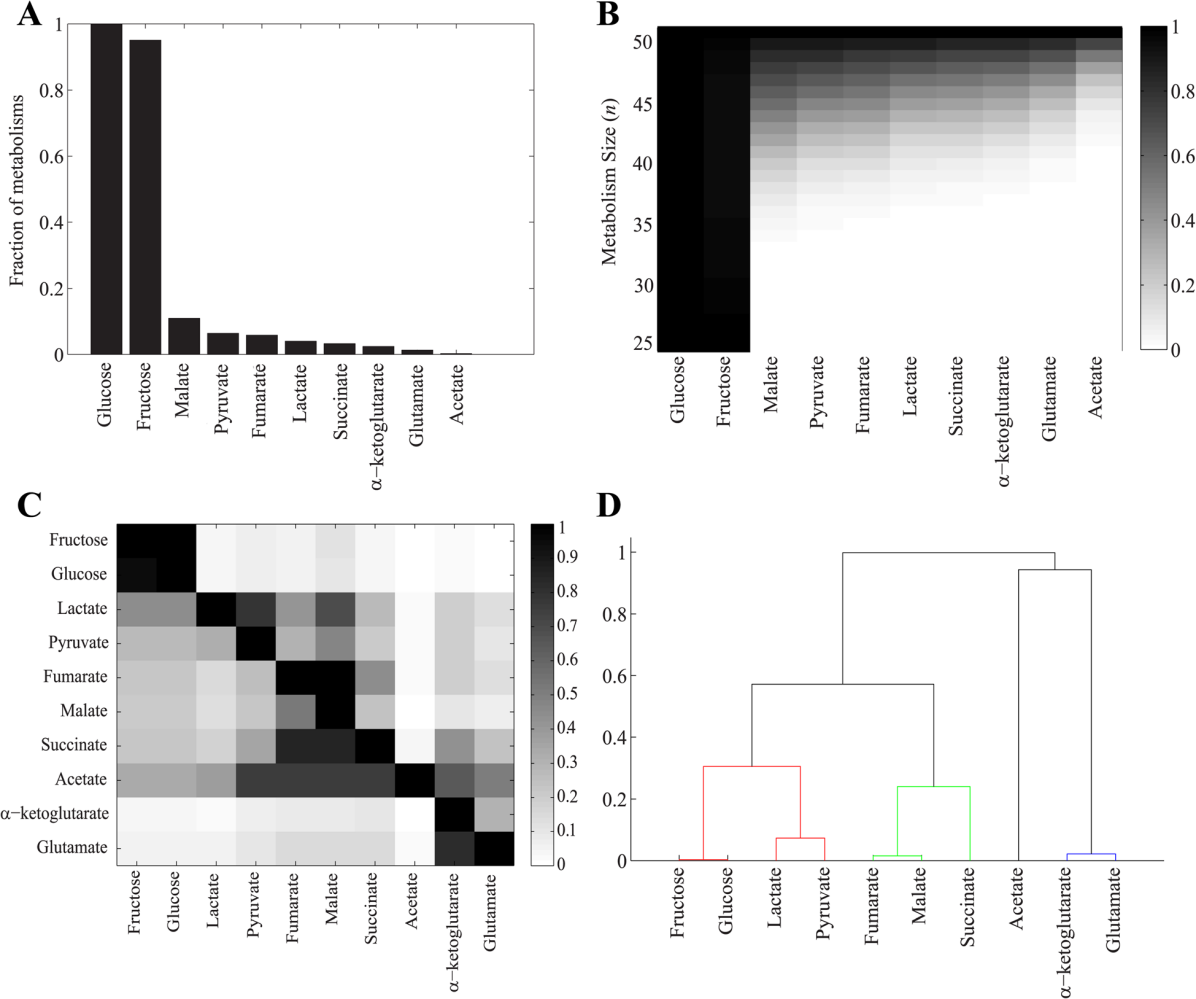
Potential for preadaptation depends on biochemical similarity between carbon sources. **a** The histogram shows the fraction of metabolisms viable on glucose as carbon source *C* that are also viable on each of the nine other carbon sources *C*_*new*_ (*x*-axis). **b** As in (**a**), but broken down by metabolism size, and fractions of viable metabolisms are represented by different shades of *grey* (see legend). **c** Fraction of metabolisms viable on carbon source *C* (*x*-axis), which are also viable on carbon source *C*_*new*_ (*y*-axis), (coded by shade of *grey*, see legend). **d** Dendrogram of carbon sources clustered based on their pairwise preadaptation propensity. We used UPGMA method (unweighted pair group method with arithmetic means) for clustering carbon sources

In a next analysis, we asked whether different carbon sources *C*_*new*_ are equally likely to occur as exaptations. Figure 4a shows, for each of nine carbon sources *C*_*new*_, and for metabolisms whose focal carbon source *C* is glucose, the fraction of metabolisms that are also viable on *C*_*new*_. The figure indicates huge disparities between carbon sources, where 97.5 % of metabolisms viable on glucose are also viable on fructose, but only 11.7 % are viable on malate, and fewer than 10 % are viable on any of the other seven carbon sources. Figure 4b shows these fractions broken down by metabolism size *n*. Almost all metabolisms viable on glucose are also viable on fructose, regardless of *n*, but the potential for preadaptation to other carbon sources is monotonically increasing with increasing *n*. Note that the only metabolism with the maximum of *n* = 51 reactions is viable on all ten carbon sources, such that at the highest *n*, the potential for preadaptation to any carbon source must reach one. These patterns are not a peculiarity of metabolisms viable on glucose, as Additional file 11 illustrates for metabolisms with lactate as the focal carbon source *C*.

Figure 4c extends this analysis to all carbon sources *C*. Its *x*-axis shows the focal carbon source *C*, its *y*-axis the carbon source *C*_*new*_, and the grey shading of each matrix entry corresponds to the fraction of metabolisms viable on *C* and *C*_*new*_. Importantly, this matrix is not symmetric, showing that the potential of preadaptation between a given pair of carbon sources is not necessarily reciprocal. For example, the probability of preadaptation to glucose among metabolisms viable on acetate is 0.33 but the probability of preadaptation to acetate among metabolisms viable on glucose is only 0.0023. Moreover, all the metabolisms viable on fumarate are also viable on malate (i.e. preadaptation probability = 1), but only 52.73 % of metabolisms viable on malate are also viable on fumarate (i.e. *preadaptation probability = 0.5273*). This asymmetry comes from the relative position of carbon sources in central carbon metabolism. For example, fumarate precedes malate in the citric acid cycle (Additional files 1 and 2), because malate is synthesized from fumarate. This ordering means that metabolisms viable on fumarate will also frequently be viable on malate, whereas the opposite is not necessarily true.

In a final analysis, we also clustered carbon sources according to their mutual propensity for preadaptation, using the hierarchical clustering method UPGMA (unweighted pair group method with arithmetic means) [43]. Figure 4d shows the resulting dendrogram. The carbon sources that cluster together are biochemically closely related, which can help explain their mutual propensity for pre-adaptation. Specifically, (i) glucose and fructose are both glycolytic carbon sources, (ii) fumarate, succinate and malate occupy consecutive steps in the citric acid cycle, (iii) pyruvate and lactate are interconvertible via lactate dehydrogenase, (iii) acetate is functionally linked to pyruvate via acetyl-coenzymeA, which is produced from pyruvate through a reaction catalyzed by pyruvate dehydrogenase, and (iv) glutamate and α-ketoglutarate are interconvertible via glutamate dehydrogenase. An analysis of metabolisms without disconnected reactions shows a similar pattern (Additional files 12 and 13). In sum, metabolisms viable on biochemically similar focal carbon sources *C* also tend to be pre-adapted to biochemically similar carbon sources *C*_*new*_ *.*

### High biomass yield and low waste production are associated with greater potential for pre-adaptation

**Fig. 5:**
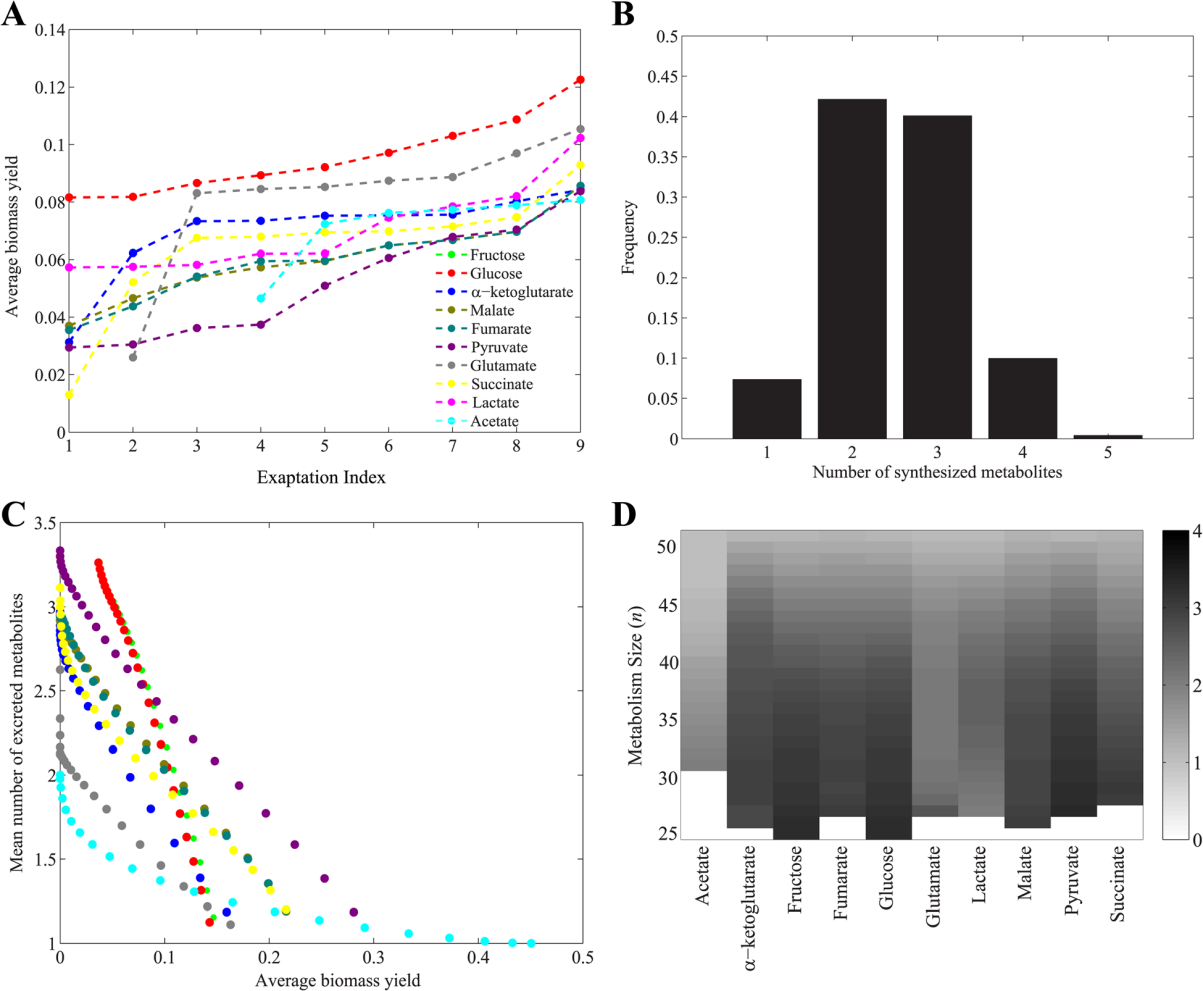
High biomass yield and low waste production are associated with greater potential for preadaptation. **a** The *x*-axis shows the exaptation index, i.e., the number of carbon sources *C*_*new*_ on which metabolisms viable on carbon source *C* (color legend) are viable. The *y*-axes show the average biomass yield. Data is based on metabolisms of size *n=40*. **b** Fraction of metabolisms excreting a given number of metabolites (*x*-axis) among metabolisms of size 40 that are viable on glucose. **c** Each circle shows the mean number of excreted metabolites (*y*-axis), and mean biomass (*x*-axis), among metabolisms of a given size that are viable on a given carbon source, colored according to the legend in (**a**). **d** Mean number of excreted metabolites (coded by shade of *grey*, see legend) among metabolisms of a given size (*y*-axis), that are viable on a given carbon source (*x*-axis). *White colors* correspond to metabolisms whose size is too small for viability on *C*

Metabolisms of the same size and that are viable on the same carbon source *C* can vary widely in their exaptation index, i.e., the number of additional carbon sources *C*_*new*_ on which they are viable. To understand the causes of this variation, we analyzed the average biomass yield per mole of carbon. We found that this yield increases with increasing exaptation index, regardless of the focal carbon source *C* (Fig. 5a), and regardless of metabolism size (Additional file 14). We also examined the number of waste metabolites, molecules that a metabolism synthesizes (and excretes) but that are not biomass precursors. This number of molecules can vary widely for metabolisms of the same size that are viable on the same carbon source *C* (Fig. 5b, and Additional file 15). Not surprisingly, metabolisms that show higher yield also excrete fewer waste molecules, regardless of their size, and regardless of their focal carbon source *C* (Fig. 5c and Additional file 16). In addition, we observed that metabolisms with more reactions generally produce less waste, regardless of their focal carbon source (Fig. 5d).

Taken together, these observations show that metabolisms with higher exaptation index are more efficient, converting more of their carbon source *C* into biomass, and excreting fewer waste products. Larger metabolisms synthesize less waste and show higher biomass yield (Additional file 17), which can help explain their greater potential for exaptation (Fig. 1b and d).

## Discussion

We systematically analyzed the potential for exaptation or preadaptation in the simple but biologically important system of central carbon metabolism. Our analysis complements a previous study based on sampling a much larger metabolic genotype space [36], because it examines the potential for exaptation in an exhaustively enumerable genotype space of 10^15^ metabolisms. Our observations are consistent with that of the previous study, in that we also find a high potential for exaptation in this smaller metabolic system.

Our central observation is that most metabolisms viable on a given carbon source *C* are also viable on one additional carbon source *C*_*new*_, and often on multiple such carbon sources. In a changing chemical environment, where the focal carbon source *C* has been consumed but where one or more carbon sources *C*_*new*_ become available, this ability can become an important innovation. In other words, potential exaptations are highly abundant, even in a simple metabolic system. They occur preferentially for carbon sources *C*_*new*_ that are biochemically similar to *C*, and are ultimately caused by shared biochemical pathways that connect different extracellular carbon sources to essential biomass precursors. Such shared pathways result in complex phenotypic correlations among different genotypes.

We also observed that different metabolisms viable on a given carbon source *C* can be preadapted to widely different sets of carbon sources *C*_*new*_. This diversity of preadaptation can help make populations robust to environmental changes in carbon source availability, because a sufficiently large and genotypically diverse population may harbor at least one metabolic genotype that is already preadapted to some newly available *C*_*new*_.

One advantage of an exhaustive enumeration approach like ours is that it allows us to systematically identify metabolic properties associated with high exaptation potential. One such property is metabolic complexity, i.e., the number of reactions in a metabolism. The greater metabolic complexity is, the greater is the number of carbon sources *C*_*new*_ on which a metabolism is viable. Another property is metabolic efficiency, the ability to convert carbon into biomass (precursors) with few waste products. The more efficient a metabolism is, the greater is its potential for exaptation. Complexity and efficiency are linked, because at least in our study system, larger metabolisms produce less waste. These associations might be more difficult to understand in genome-scale systems with thousands of reactions, but studying them in a simpler system leads to a straightforward explanation. Specifically, in a larger metabolism it is more likely that most reactions link carbon sources and biomass productively, without producing dead-end products that cannot be used by other reactions. And this very feature makes it also more likely that a reaction path exists from any one carbon source *C*_*new*_ to each biomass precursor.

Among the limitations of our study is that we focused on carbon metabolism, and the metabolism of other chemical elements, such as nitrogen or sulfur, may differ in its exaptation potential. To find out whether this is the case remains a task for future work, but we note that sulfur and nitrogen metabolism generally show similar properties to carbon metabolism in studies of metabolic genotype spaces [44, 45].

A second limitation is that our analysis focuses on the presence or absence of reactions, and it neglects regulatory constraints arising through sub-optimal expression or regulation of an enzyme. This is consistent with our focus on the qualitative feature of viability, for which the presence or absence of reactions (enzymes) is more important than their quantitative regulation. We also note that regulatory constraints can be readily broken in evolution, even on the short time scales of laboratory evolution experiments [46–48].

A third limitation comes from our reaction-centered definition of metabolic genotypes. This coarse-grained definition of metabolic genotype is widely used [21–24, 27, 28], because it is simple, computationally efficient, and yet sufficiently informative for many analyses. However, it neglects that there need not be a one-to-one relationship between metabolic genes and metabolic reactions. Some reactions are catalyzed by multiple enzymes [49], and some enzymes catalyze multiple reactions [50–52]. One recent study that speaks to this limitation has focused on promiscuous enzymes that catalyze multiple biochemical reactions in genome-scale metabolisms. It shows that considering this one-to-many relationship between enzymes and reactions leads to an increase in the number of environments in which a metabolism is viable [53]. This would also apply to our study system, because adding reactions catalyzed by promiscuous enzymes to a metabolism can only increase its potential for exaptation. That is, addition of a reaction cannot abolish viability on the focal carbon source, but it might convey the ability to survive on further carbon sources *C*_*new*_.

Fourth, while *E.coli* central carbon metabolism is more complete than that of other species, where, for example, parts of the citric acid cycle are missing [42], we have not considered all reactions that could be considered part of a central carbon metabolism. For example, we have only considered the canonical Embden-Meyerhof-Parnas glycolytic pathway, and we have neglected reactions belonging to the Entner-Doudoroff (ED) and the phosphoketolase pathways [54, 55]. This was necessary, because the size of the genotype space we analyze is already large (10^15^ genotypes), and at the limit of feasibility for exhaustive genotype-phenotype mapping [40, 41]. Any additional reactions would have made an exhaustive analysis impossible. Just as for the preceding limitation, we note that adding these or other reactions to any one metabolism could only increase its potential for exaptation. For this reason, considering more complex metabolisms would not affect our core observation that many variants of central carbon metabolism harbor exaptive potential.

Finally, we only considered ten carbon sources, whereas metabolic generalists like *E.coli* can be viable on many more carbon sources [56, 57]. However, even this low number of carbon sources was sufficient to detect a high potential for exaptation, and once again, considering more carbon sources could only increase the estimated exaptive potential.

One can envision the following evolutionary scenario in which traits with exaptive potential facilitate survival in novel environments. Consider a minimal metabolic network adapted to a specific carbon source *C*, i.e., a network from which no reaction can be removed without abolishing viability on *C*. Many such minimal networks are also viable on one or more additional carbon sources *C*_*new*_. If *C*_*new*_ becomes available and the organism hosting this metabolism (or its competitors) has consumed *C*, then viability on *C*_*new*_ becomes an adaptation that helps the organism survive. The survivor’s descendants undergo processes such as gene duplication, point mutations, and horizontal gene transfer, which may enable some of them to catalyze novel reactions that allow it to utilize a carbon source *C’* (and as a by-product, perhaps one or more additional carbon sources *C’*_*new*_). If none of these carbon sources ever occur in the environment, any such genetic change will eventually disappear through genetic drift or degenerative mutations. However, if *C’* occurs in the environment, a new adaptation with exaptive potential on carbon source *C’*_*new*_ has arisen. In other words, one can envision a step-wise expansion of metabolism that is driven by adaptive processes, but in which the exaptive potential of some traits facilitates survival in novel environments. That different genotypes viable on *C* are viable on different carbon sources *C*_*new*_ (Fig. 3) may further facilitate adaptive evolution.

The high exaptation potential of central carbon metabolism, and of genome-scale metabolisms in general [36] invites speculation that many metabolic innovations originate non-adaptively. However, we emphasize that our analysis is not suited to identify any one metabolic trait as an exaptation. It can thus also not identify the incidence of exaptations in metabolic evolution, which remains a major challenge for future work.

## Conclusion

We analyzed central carbon metabolism, a metabolic system small enough to lend itself to exhaustive genotype-phenotype mapping, and have systematically quantified this system’s potential for preadaptation for viability on novel carbon sources. Our results indicate that metabolisms viable on any one carbon source can be preadapted to multiple other carbon sources. The potential for such preadaptation rises with the complexity of a metabolism, i.e., with its numbers of reactions, and with its efficiency. It results from correlations between the phenotypes of different genotypes, which are caused by shared pathways that connect different extracellular carbon sources to essential biomass precursors.

## Methods

### Flux balance analysis

Flux Balance Analysis (FBA) is a widely used computational method that predicts the metabolic flux through biochemical reactions in metabolic networks [58–61]. FBA uses information about the stoichiometric coefficients of the metabolites participating in each reaction, encapsulated in the stoichiometric matrix *S*, which is of dimension *m*×*n,* where *m* and *n*, respectively, denote the number of metabolites and the number of reactions in a metabolism. FBA assumes that a metabolism has reached a steady-state, as might be attained by a growing population of bacteria in chemostat with constant nutrient supply, where mass conservation constraints apply. These constraints are mathematically expressed as *Sv* = 0, where *v* is the vector of fluxes (*v*_*i*_) through reaction *i*. The solution space of this equation is called the null space of the stoichiometric matrix (*S*). This null space is further constrained by upper and lower bounds on the fluxes through each reaction. FBA applies linear programming to find the optimal flux vector(s) that maximize an objective function Z. This task can be mathematically formulated as finding a flux vector *(v* ^*^ *)* with the property

$$ {v}^{*}=ma{x}_vZ(v)=\left\{{c}^Tv\Big|S.v=0,\ a\le v\le b\right\}, $$

where the vector *c* contains scalar coefficients representing a maximization criterion, and entries *a*_*i*_ and *b*_*i*_ of vectors *a* and *b*, respectively, indicate the minimally and maximally possible flux through reaction *i*.

We use a set of 13 well-known precursors from central carbon metabolism as biomass molecules required for viability (Additional file 2: Table S1). We use the software package CLP (1.4, Coin-OR; https://projects.coin-or.org/Clp) to solve all linear programming problems.

### Chemical environments and carbon sources

To computationally predict the viability of a metabolism on a given carbon source, information about the chemical environment that contains this and other nutrients needed to synthesize biomass precursors is required. In our analysis of central carbon metabolism, we consider a minimal aerobic growth environment composed of a sole carbon source, along with ammonium as a nitrogen source, inorganic phosphate as a source of phosphorus, as well as oxygen, protons, and water. Different environments vary in their carbon source but are the same in other nutrients. A metabolism is viable on a given carbon source, if it can synthesize all 13 biomass precursors (Additional files 1 and 2) from that carbon source. In our study, we used the following carbon sources: D-glucose, acetate, pyruvate, D-lactate, D-fructose, alpha-ketoglutarate, fumarate, malate, succinate and glutamate.

### Reaction universe

As a reaction “universe” we use a global set of reactions in central carbon metabolism, which is based on a published reconstruction of *E. coli* central carbon metabolism [29]. From this reconstruction, we deleted four reactions involved in ethanol metabolism, because in this study we are not interested in ethanol biosynthesis or degradation. We also grouped the reactions catalyzed by aconitase A and aconitase B into one reaction. The final reaction set consists of *N* = 51 intracellular reactions (Additional files 1 and 2). The reconstruction in [29] also involves 20 transport reactions, which are necessary to import nutrients or excrete waste products, and which we assume to be present in all metabolisms we study.

### Genotype-phenotype mapping in metabolic genotype space

For computational expediency, we use a compact representation of a metabolic genotype that is based on reactions rather than genes. Specifically, we represent such a genotype as a binary vector whose *i*-*th* entry corresponds to the *i-th* reaction in our reaction universe. If an organism’s genome encodes an enzyme capable of catalyzing a given reaction, the corresponding entry in the genotype vector will be one and zero otherwise. The genotype space including all possible metabolisms comprises 2^*N*^ metabolisms, where *N* is the total number of known or considered chemical reactions (*N* = 51 for our analysis). Any metabolic genotype can be thought of as a point in this space. We consider a metabolism viable on a carbon source if its biomass synthesis rate is more than 1 % of the biomass synthesis rate of the network formed by all *N*=51 reactions in central carbon metabolism. Some metabolic genotypes correspond to metabolisms in which some reactions are disconnected.

We call a reaction disconnected if (*i*) its products are neither biomass precursors nor substrates of any other reaction of the metabolism, or (*ii*) at least one of its substrates is neither a product of other reactions nor a nutrient taken up from the environment. We performed some analyses separately for metabolisms with and without disconnected reactions, to find out whether the presence of such reactions would affect our conclusions.

### Exhaustive enumeration of viable metabolisms

To exhaustively characterize the phenotypes of all 2^51^ (10^15^) metabolic genotypes would be prohibitive if one had to perform one FBA computation (consuming about 10^−2^ seconds) for each genotype. However, this computation becomes feasible when two facts are considered [39, 40]. First, six among the 51 internal reactions of central carbon metabolism are essential for viability on every carbon source we consider [62], which reduces the number of required FBA computations by a factor 2^6^ from 2^51^ (10^15^) to 2^45^ (10^13^). Second, deleting one or more reactions from an inviable metabolism cannot result in a viable metabolism, such that all metabolisms (“children”) that contain a subset of the reactions of an inviable metabolism (“parent”) will also be inviable. An algorithm that takes this observation into account decreases the number of required FBA evaluations further to approximately 10^9^ [41].

The set of metabolisms that are viable on a given subset *S* of the ten carbon sources can easily be identified after the set *GN*(*C*_*i*_) of metabolisms viable on each of the ten carbon sources *C*_*i*_ has been determined. Specifically, *V*(*S*) = {*G* ∈ Ω, ∀ *C*_*i*_ ∈ *S*, ∀ *C*_*j*_ ∈ *S* ^'^|*G* ∈ *GN*(*C*_*i*_), *G* ∉ *GN*(*C*_*j*_)} where *G* denotes a genotype from genotype space *Ω*, and *S’* denotes the complement of *S*.

## Additional files

Additional file 1: Central carbon metabolism. Each arrow in each panel corresponds to one of the 51 internal reactions we consider. Metabolites are indicated by their acronyms (see Additional file 2). Boxed metabolites correspond to 13 essential biomass precursors. Note that 4 metabolites (accoa, g3p, f6p and e4p) are shown more than once for visual clarity. Metabolic pathways, including glycolysis/gluconeogenesis, pentose-phosphate pathway, citric-acid cycle, oxidative phosphorylation, pyruvate and glutamate metabolism are distinguished by the colored and dashed rectangles. Anaplerotic reactions and glyoxylate shunt are highlighted using the purple and green arrows respectively. The figure is taken by permission from [40]. (TIF 2163 kb)
Additional file 2: Metabolites and reactions in central carbon metabolism. This table lists all the reactions (Sheet 1) and metabolites (Sheet 2) of central carbon metabolism, their abbreviations, and further associated information. (XLSX 18 kb)
Additional file 3: Metabolisms viable on a given carbon source vary widely in their exaptation potential. Histogram of the exaptation index (*x*-axis), i.e., the number of carbon sources *C*_*new*_ on which a metabolism is viable, for metabolisms viable on lactate as carbon source *C* with size (A) 35, (B) 40, (C) 45, and for metabolisms viable on malate as carbon source *C* with size (D) 35, (E) 40, (F) 45. (TIF 496 kb)
Additional file 4: High exaptation potential in central carbon metabolisms (considering only metabolisms without disconnected reactions). (A) Fraction of metabolisms with exaptation index *I>0* (*y*-axis) viable on some carbon source *C* (*x*-axis). Red bars correspond to viable metabolisms without disconnected reactions, and blue bars correspond to all viable metabolisms. (B) Fraction of metabolisms (coded by shade of grey, see legend) with exaptation index (*I>0*) that are viable on some carbon source *C* (*x*-axis) and have a given size (*y*-axis), (C) Mean exaptation index of metabolisms without disconnected reactions, and viable on a given focal carbon source *C* (*x*-axis). Red bars correspond to viable metabolisms without disconnected reactions, and blue bars correspond to all viable metabolisms. (D) Mean exaptation index (coded by shade of grey, see legend) of metabolisms without disconnected reactions, and viable on some carbon source *C* (*x*-axis) and with a given size (*y*-axis). White colors in (B) and (D) correspond to metabolisms whose size is too small for viability on *C*. (TIF 590 kb)
Additional file 5: Exaptation diversity. For metabolisms whose focal carbon source *C* is shown on the *x*-axis, and the number of reactions (*n*) is shown on the vertical axis, the number of carbon sources *C*_*new*_ on which at least one metabolism is preadapted, is coded by shade of grey (see legend). (TIF 100 kb)
Additional file 6: Metabolisms viable on glucose as the main carbon source *C* can differ greatly in their viability on other carbon sources. The figure shows a histogram of the phenotype distance (*x*-axis), for metabolisms of size (A) 35, and (B) 40, viable on glucose as carbon source *C*. (TIF 226 kb)
Additional file 7: Metabolisms viable on pyruvate as the main carbon source *C* can differ greatly in their viability on other carbon sources. The figure shows a histogram of the phenotype distance (*x*-axis), for metabolisms of size (A) 30, (B) 35, (C) 40, and (D) 45, viable on pyruvate as focal carbon source C. (TIF 417 kb)
Additional file 8: Metabolisms can preadapt to a wide variety of carbon sources (considering only metabolisms without disconnected reactions). (A) For metabolisms (without disconnected reactions) whose focal carbon source *C* is shown on the *x*-axis, and the number of reactions (*n*) is shown on the vertical axis, each shade of grey (see legend) shows the number of carbon sources *C*_*new*_ on which at least one metabolism is preadapted. (B) Mean phenotypic distance (see legend) of metabolisms (without disconnected reactions) viable on a focal carbon source (*x*-axis) and with a given number of reactions *n* (*y*-axis). (TIF 219 kb)
Additional file 9: Metabolisms viable on glucose as the main carbon source *C* can differ greatly in their viability on other carbon sources (considering only metabolisms without disconnected reactions). The figure shows a histogram of the phenotype distance (*x*-axis), for metabolisms without disconnected reactions with size (A) 30, (B) 35, (C) 40, and (D) 45, viable on glucose as carbon source *C*. (TIF 427 kb)
Additional file 10: Metabolisms viable on pyruvate as the main carbon source *C* can differ greatly in their viability on other carbon sources (considering only metabolisms without disconnected reactions). The figure shows a histogram of the phenotype distance (*x*-axis), for metabolisms without disconnected reactions with size (A) 30, (B) 35, (C) 40, and (D) 45, viable on pyruvate as carbon source *C*. (TIF 428 kb)
Additional file 11: Metabolisms viable on lactate differ in their propensity for preadaptation to other carbon sources *C*_*new*_. (A) The histogram shows the fraction of metabolisms viable on lactate as carbon source *C* that are also viable on each of the nine other carbon sources *C*_*new*_ (*x*-axis). (B) As in (A), but broken down by metabolism size, and fractions of viable metabolisms are coded by shade of grey, see legend. (TIF 218 kb)
Additional file 12: Potential for preadaptation depends on biochemical similarity between carbon sources (considering only metabolisms without disconnected reactions). (A) The histogram shows the fraction of metabolisms (without disconnected reactions) viable on glucose as carbon source *C* that are also viable on each of the nine other carbon sources *C*_*new*_ (*x*-axis). (B) As in (A), but broken down by metabolism size, and fractions of viable metabolisms are coded by shade of grey, see legend (C) Fraction of metabolisms (without disconnected reactions) viable on carbon source *C* (*x*-axis), that are also viable on carbon source *C*_*new*_ (*y*-axis), are coded by shade of grey, see legend. (D) Dendrogram of carbon sources clustered based on their pairwise preadaptation propensity. We used UPGMA method (unweighted pair group method with arithmetic means), for clustering carbon sources. (TIF 472 kb)
Additional file 13: Metabolisms viable on lactate have different potential for preadaptation to other carbon sources *C*_*new*_ (considering only metabolisms without disconnected reactions). (A) The histogram shows the fraction of metabolisms (without disconnected reactions) viable on lactate as carbon source *C* that are also viable on each of the nine other carbon sources *C*_*new*_ (*x*-axis). (B) As in (A), but broken down by metabolism size, and fractions of viable metabolisms are coded by shade of grey, see legend. (TIF 222 kb)
Additional file 14: Association between exaptation potential and biomass yield. The *x*-axes show the exaptation index, i.e., the number of carbon sources *C*_*new*_ on which metabolisms viable on carbon source *C* (color legend) are viable. The *y*-axes show the average biomass yield. Data is based on metabolisms of size (A) *n=35*, and (B) *n=45*. (TIF 334 kb)
Additional file 15: Distribution of the number of synthesized metabolites among metabolisms viable on glucose. Fraction of metabolisms excreting a given number of metabolites (*x*-axis) among metabolisms viable on glucose with size (A) *n=35*, and (B) *n=45*. (TIF 174 kb)
Additional file 16: Association between biomass yield and the number of excreted waste metabolites. The vertical axis shows the biomass yield of metabolisms of size (A) *n=30*, (B) *n=40*, and (C) *n=50* viable on glucose that excrete a given number of metabolites (*x*-axis). Boxes span the *25*-th to *75*-th percentile, and whiskers indicate the maximum and minimum values. (TIF 207 kb)
Additional file 17: Larger metabolisms have higher biomass yield. Mean of biomass yield among metabolisms of a given size (*y*-axis), that are viable on a given carbon source (*x*-axis), coded by shade of grey, see legend. White colors correspond to metabolisms whose size is too small for viability on *C*. (TIF 116 kb)

## Abbreviations

accoa: acetyl coenzyme A
ATP: Adenosine triphosphate
*C*: Carbon source
CLP: Coin-or linear progamming solver
*C*_*new*_: New carbon source
e4p: erythrose 4-phosphate
f6p: fructose 6-phosphate
FBA: Flux Balance Analysis
G: Genotype
g3p: glyceraldehyde 3-phosphate
GN: Set of viable genotypes
I: Exaptation index
n: number of reactions
NADH: Nicotinamide adenine dinucleotide
NADPH: Nicotinamide adenine dinucleotide phosphate
PP: Pentose phosphate pathway
TCA: Tricarboxylic acid cycle
UPGMA: Unweigthed pair group method with arithmetic means
Z: Objective function
