# Supplementary figures and images for: The potential for non-adaptive origins of evolutionary innovations in central carbon metabolism
Source: BMC Syst Biol. 2016 Oct 21;10:97. doi: 10.1186/s12918-016-0343-7 (PMC5073748; doi:10.1186/s12918-016-0343-7)

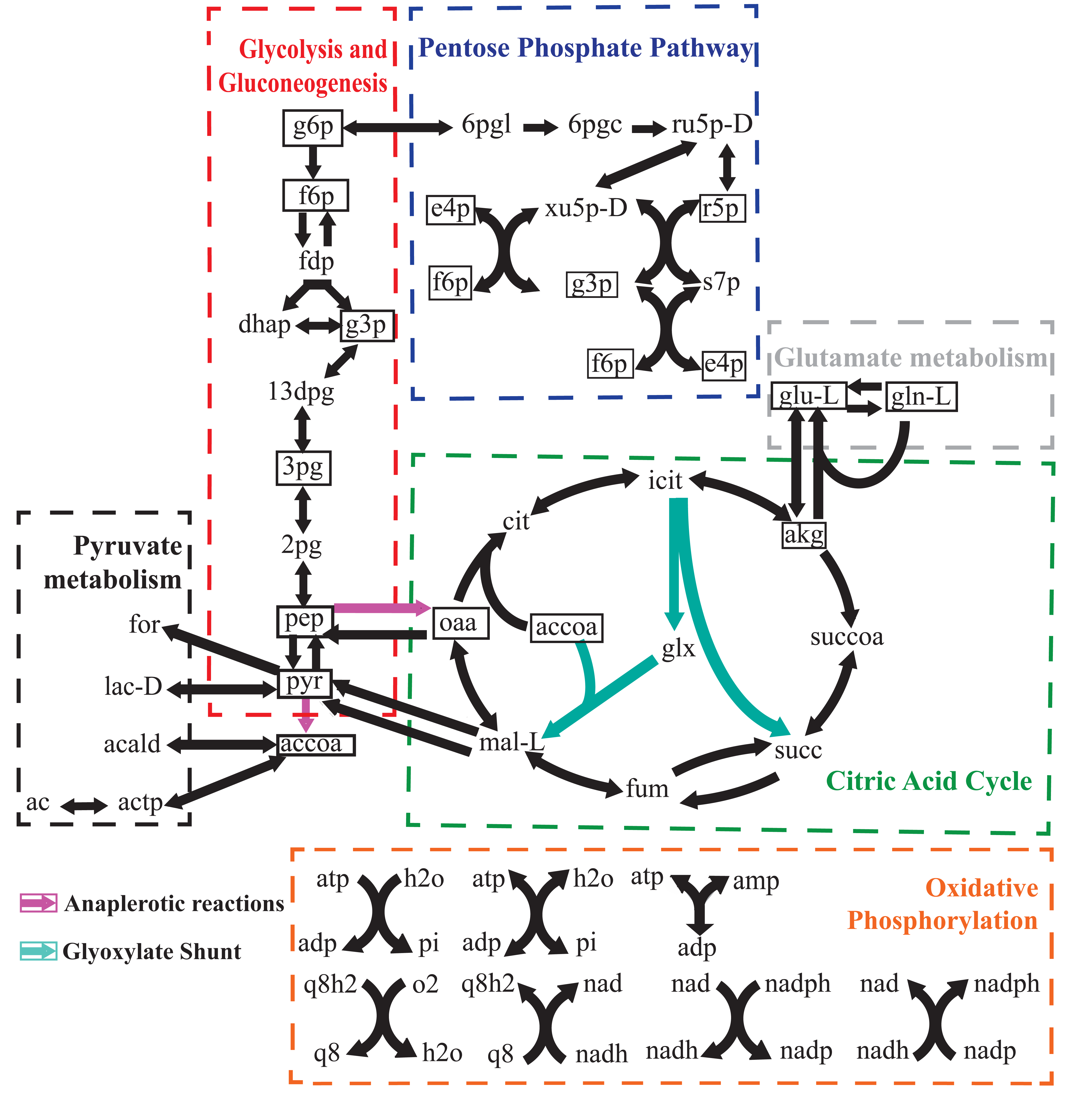

Supplement: Additional file 1: — Central carbon metabolism. Each arrow in each panel corresponds to one of the 51 internal reactions we consider. Metabolites are indicated by their acronyms (see Additional file 2). Boxed metabolites correspond to 13 essential biomass precursors. Note that 4 metabolites (accoa, g3p, f6p and e4p) are shown more than once for visual clarity. Metabolic pathways, including glycolysis/gluconeogenesis, pentose-phosphate pathway, citric-acid cycle, oxidative phosphorylation, pyruvate and glutamate metabolism are distinguished by the colored and dashed rectangles. Anaplerotic reactions and glyoxylate shunt are highlighted using the purple and green arrows respectively. The figure is taken by permission from [40]. (TIF 2163 kb) [file 12918_2016_343_MOESM1_ESM.tif]

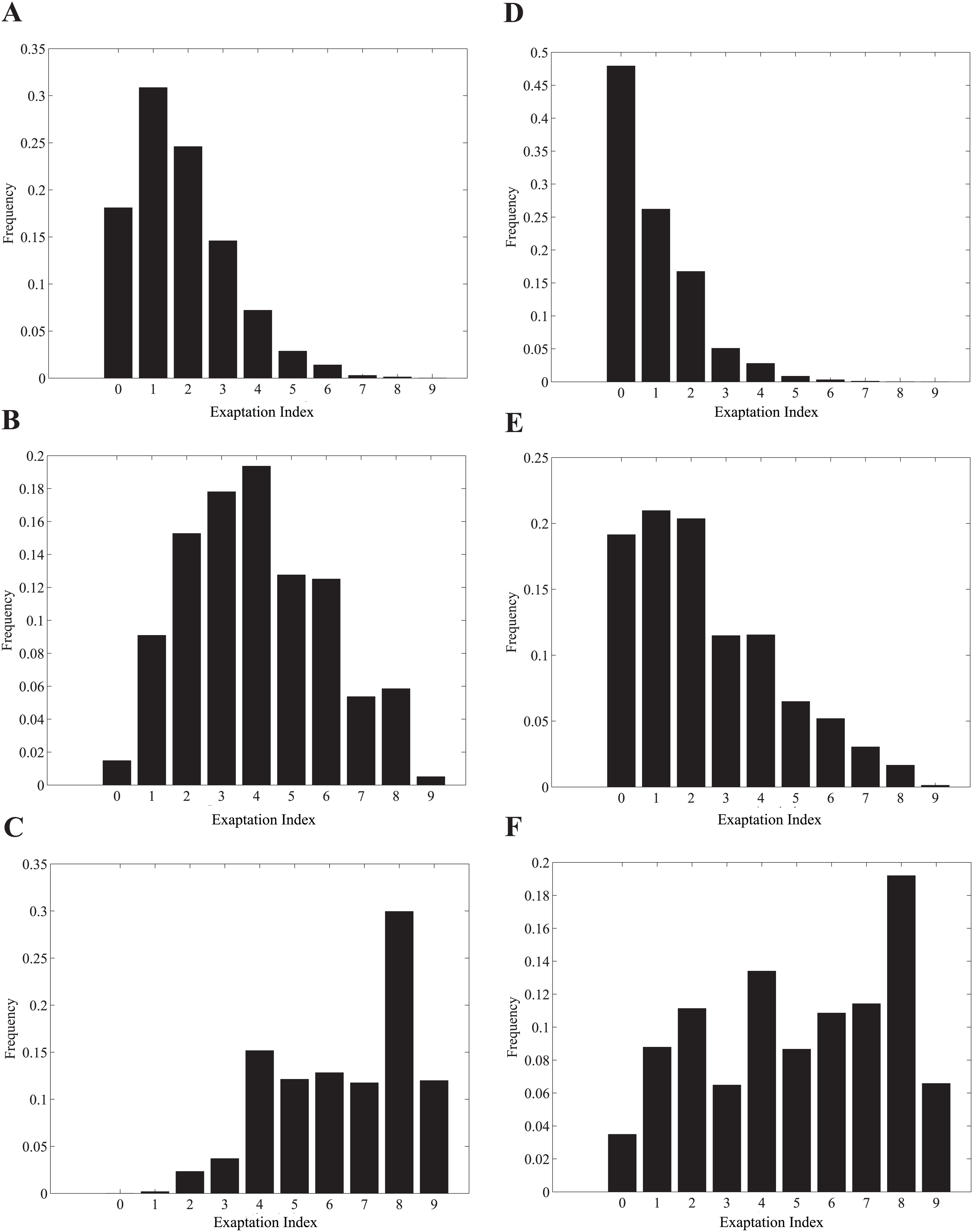

Supplement: Additional file 3: — Metabolisms viable on a given carbon source vary widely in their exaptation potential. Histogram of the exaptation index (x-axis), i.e., the number of carbon sources C new on which a metabolism is viable, for metabolisms viable on lactate as carbon source C with size (A) 35, (B) 40, (C) 45, and for metabolisms viable on malate as carbon source C with size (D) 35, (E) 40, (F) 45. (TIF 496 kb) [file 12918_2016_343_MOESM3_ESM.tif]

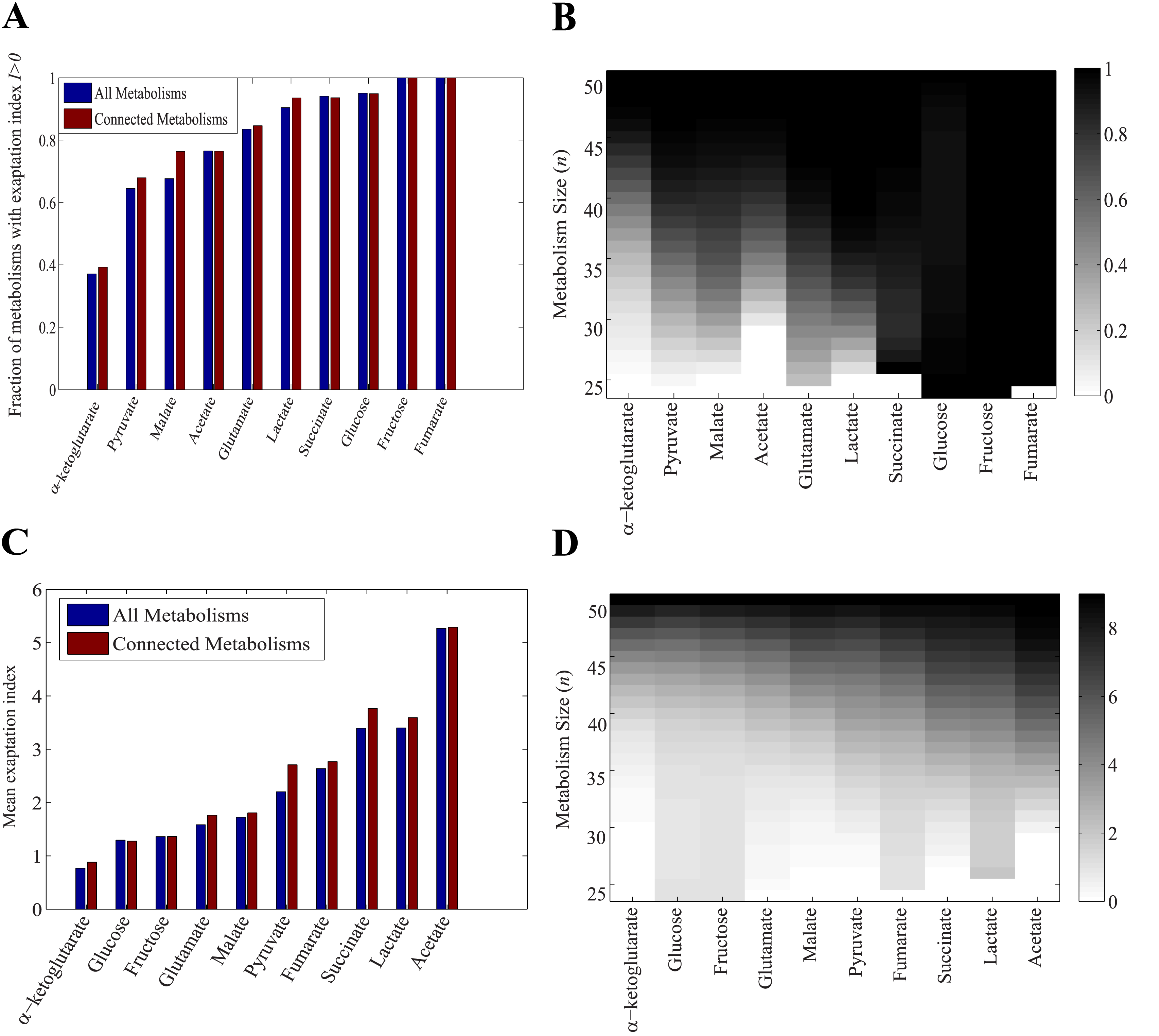

Supplement: Additional file 4: — High exaptation potential in central carbon metabolisms (considering only metabolisms without disconnected reactions). (A) Fraction of metabolisms with exaptation index I>0 (y-axis) viable on some carbon source C (x-axis). Red bars correspond to viable metabolisms without disconnected reactions, and blue bars correspond to all viable metabolisms. (B) Fraction of metabolisms (coded by shade of grey, see legend) with exaptation index (I>0) that are viable on some carbon source C (x-axis) and have a given size (y-axis), (C) Mean exaptation index of metabolisms without disconnected reactions, and viable on a given focal carbon source C (x-axis). Red bars correspond to viable metabolisms without disconnected reactions, and blue bars correspond to all viable metabolisms. (D) Mean exaptation index (coded by shade of grey, see legend) of metabolisms without disconnected reactions, and viable on some carbon source C (x-axis) and with a given size (y-axis). White colors in (B) and (D) correspond to metabolisms whose size is too small for viability on C. (TIF 590 kb) [file 12918_2016_343_MOESM4_ESM.tif]

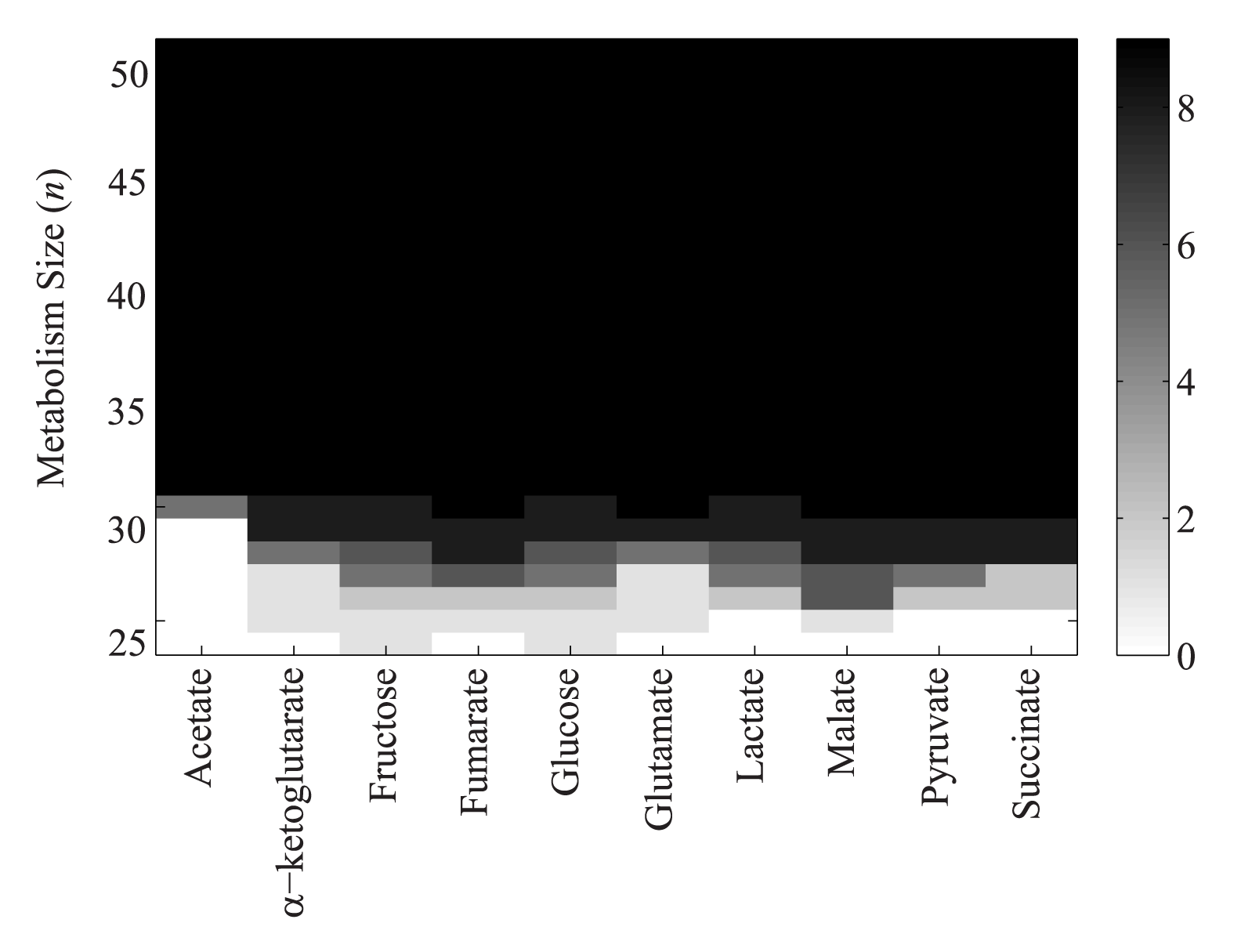

Supplement: Additional file 5: — Exaptation diversity. For metabolisms whose focal carbon source C is shown on the x-axis, and the number of reactions (n) is shown on the vertical axis, the number of carbon sources C new on which at least one metabolism is preadapted, is coded by shade of grey (see legend). (TIF 100 kb) [file 12918_2016_343_MOESM5_ESM.tif]

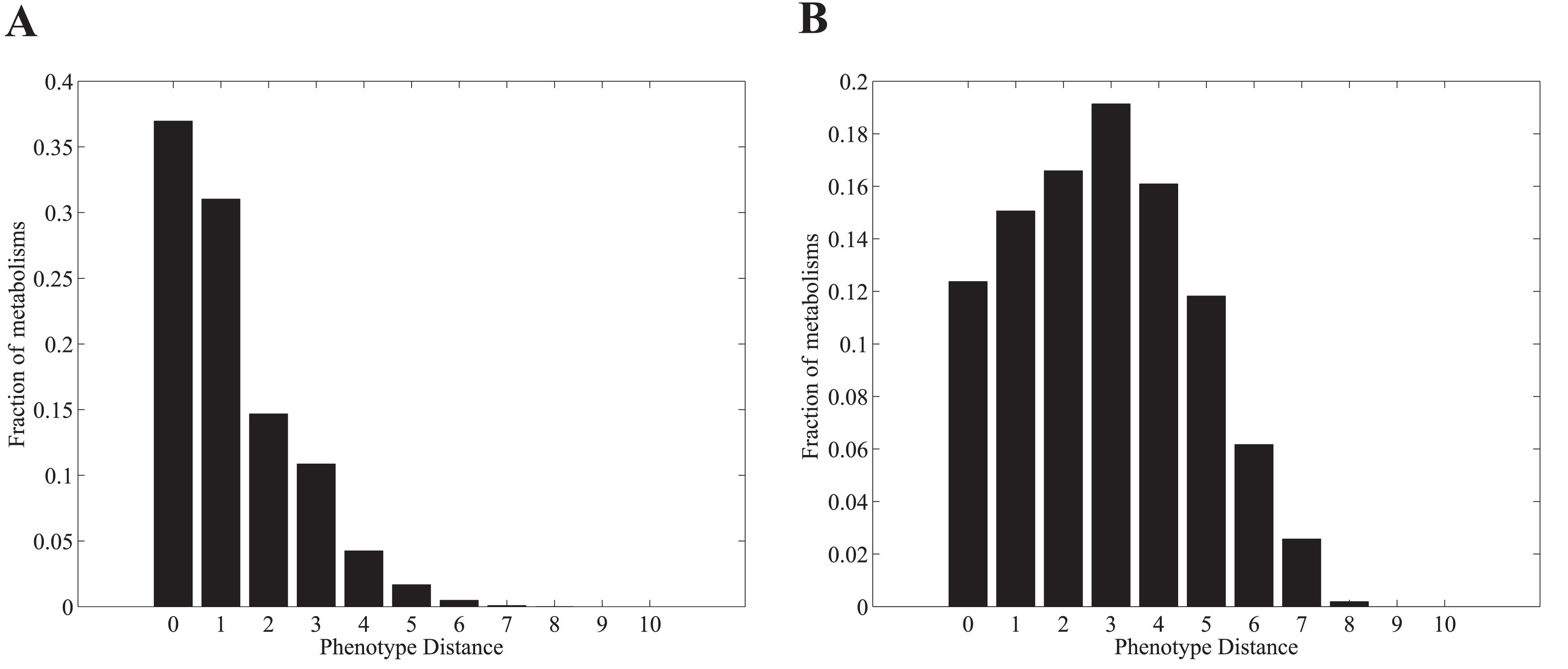

Supplement: Additional file 6: — Metabolisms viable on glucose as the main carbon source C can differ greatly in their viability on other carbon sources. The figure shows a histogram of the phenotype distance (x-axis), for metabolisms of size (A) 35, and (B) 40, viable on glucose as carbon source C. (TIF 226 kb) [file 12918_2016_343_MOESM6_ESM.tif]

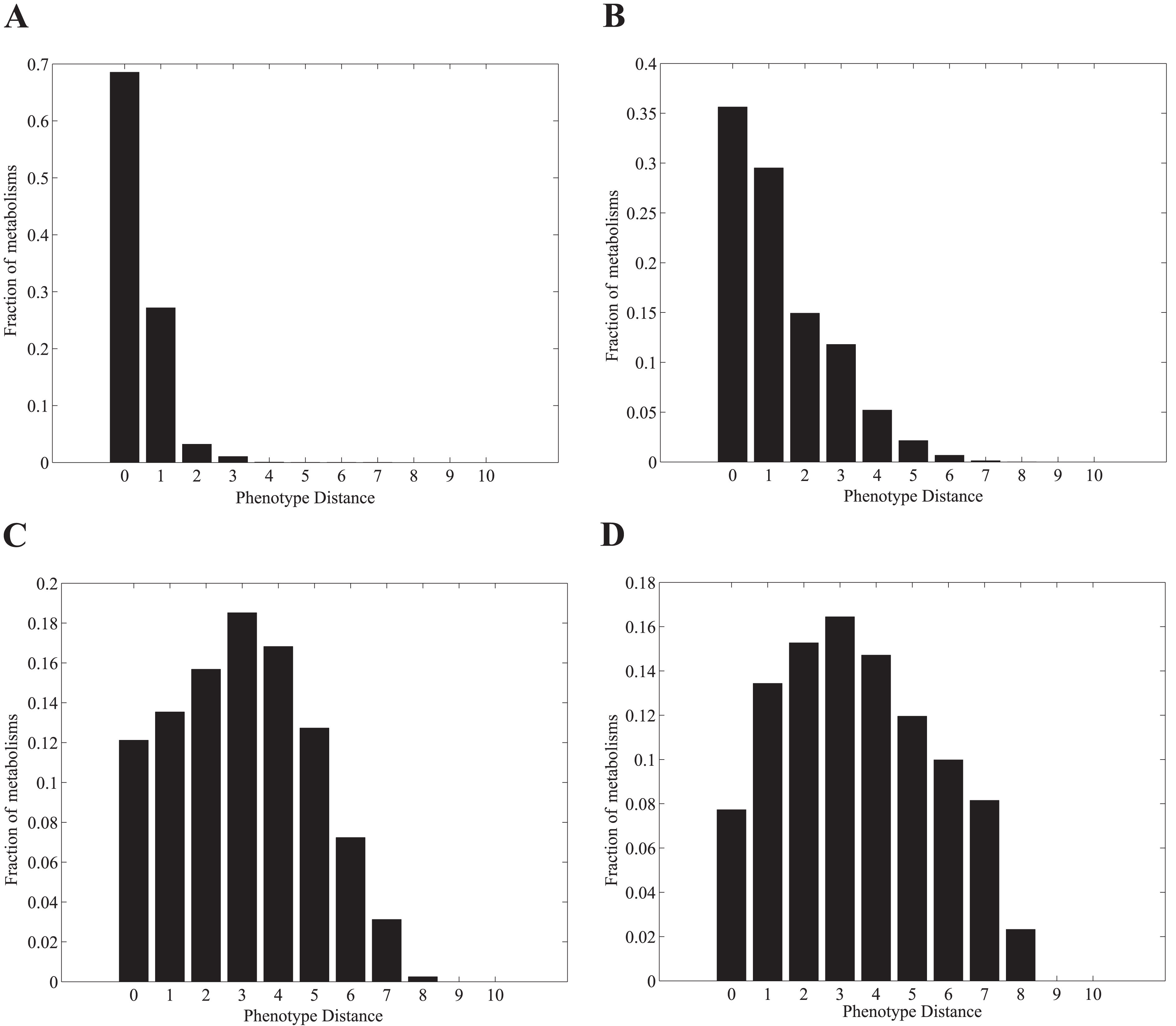

Supplement: Additional file 7: — Metabolisms viable on pyruvate as the main carbon source C can differ greatly in their viability on other carbon sources. The figure shows a histogram of the phenotype distance (x-axis), for metabolisms of size (A) 30, (B) 35, (C) 40, and (D) 45, viable on pyruvate as focal carbon source C. (TIF 417 kb) [file 12918_2016_343_MOESM7_ESM.tif]

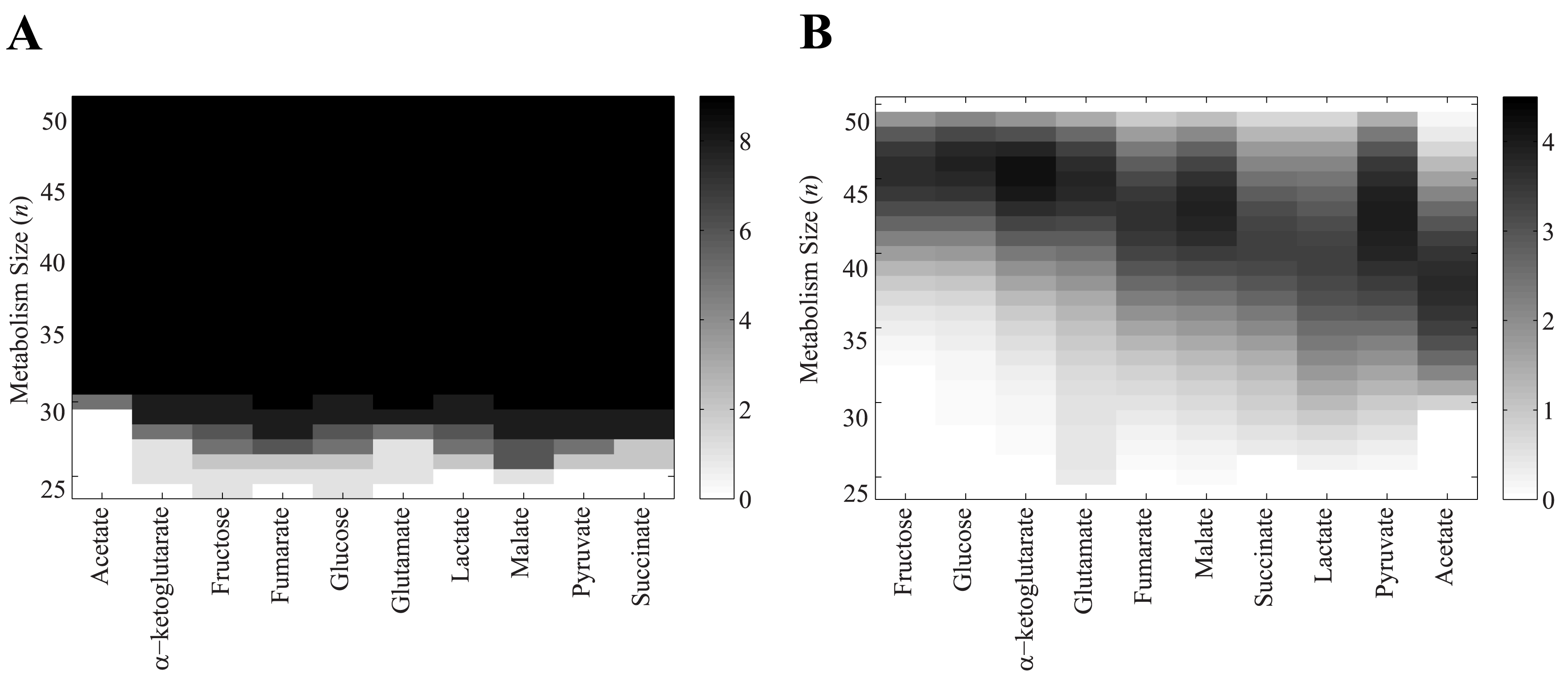

Supplement: Additional file 8: — Metabolisms can preadapt to a wide variety of carbon sources (considering only metabolisms without disconnected reactions). (A) For metabolisms (without disconnected reactions) whose focal carbon source C is shown on the x-axis, and the number of reactions (n) is shown on the vertical axis, each shade of grey (see legend) shows the number of carbon sources C new on which at least one metabolism is preadapted. (B) Mean phenotypic distance (see legend) of metabolisms (without disconnected reactions) viable on a focal carbon source (x-axis) and with a given number of reactions n (y-axis). (TIF 219 kb) [file 12918_2016_343_MOESM8_ESM.tif]

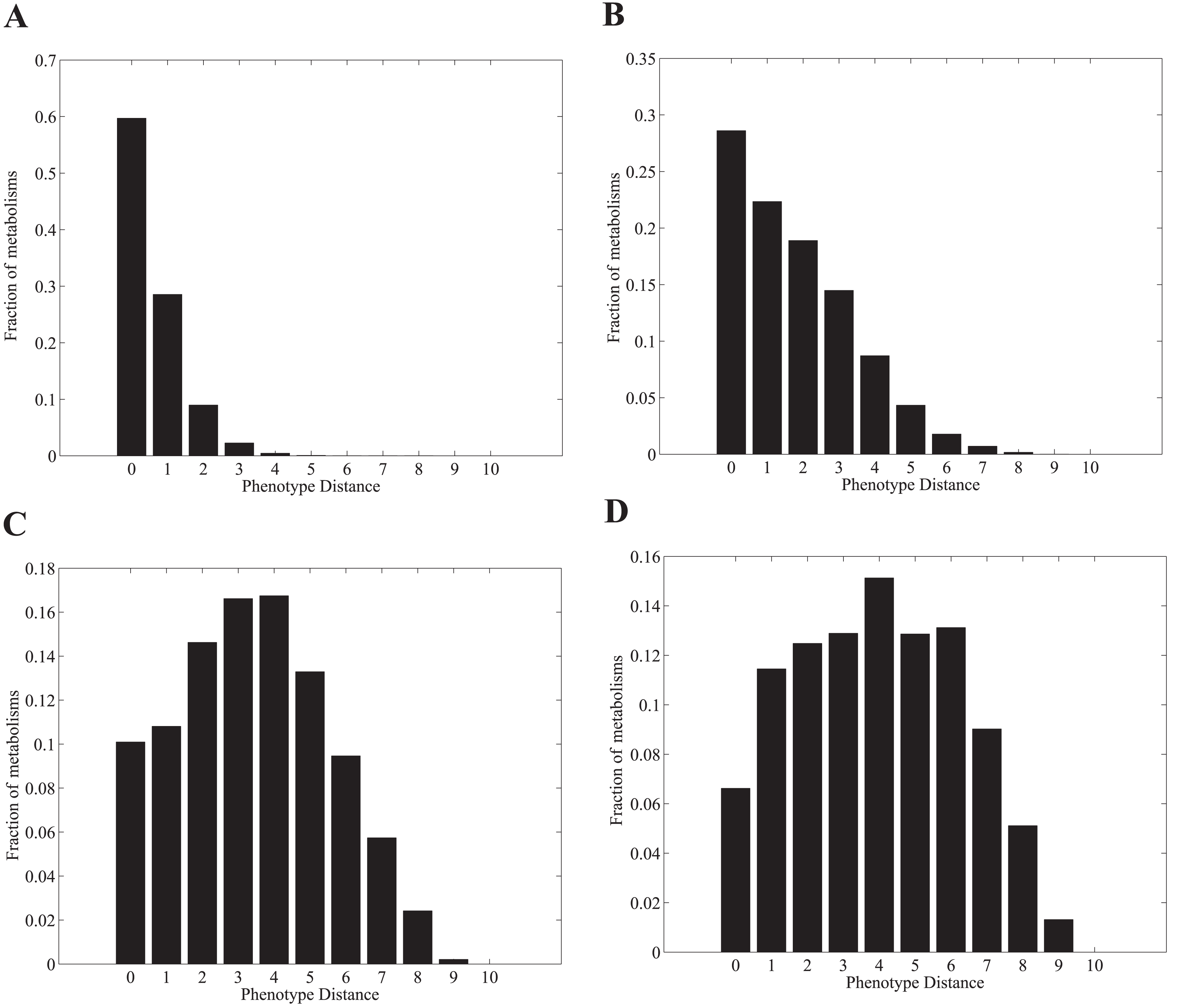

Supplement: Additional file 9: — Metabolisms viable on glucose as the main carbon source C can differ greatly in their viability on other carbon sources (considering only metabolisms without disconnected reactions). The figure shows a histogram of the phenotype distance (x-axis), for metabolisms without disconnected reactions with size (A) 30, (B) 35, (C) 40, and (D) 45, viable on glucose as carbon source C. (TIF 427 kb) [file 12918_2016_343_MOESM9_ESM.tif]

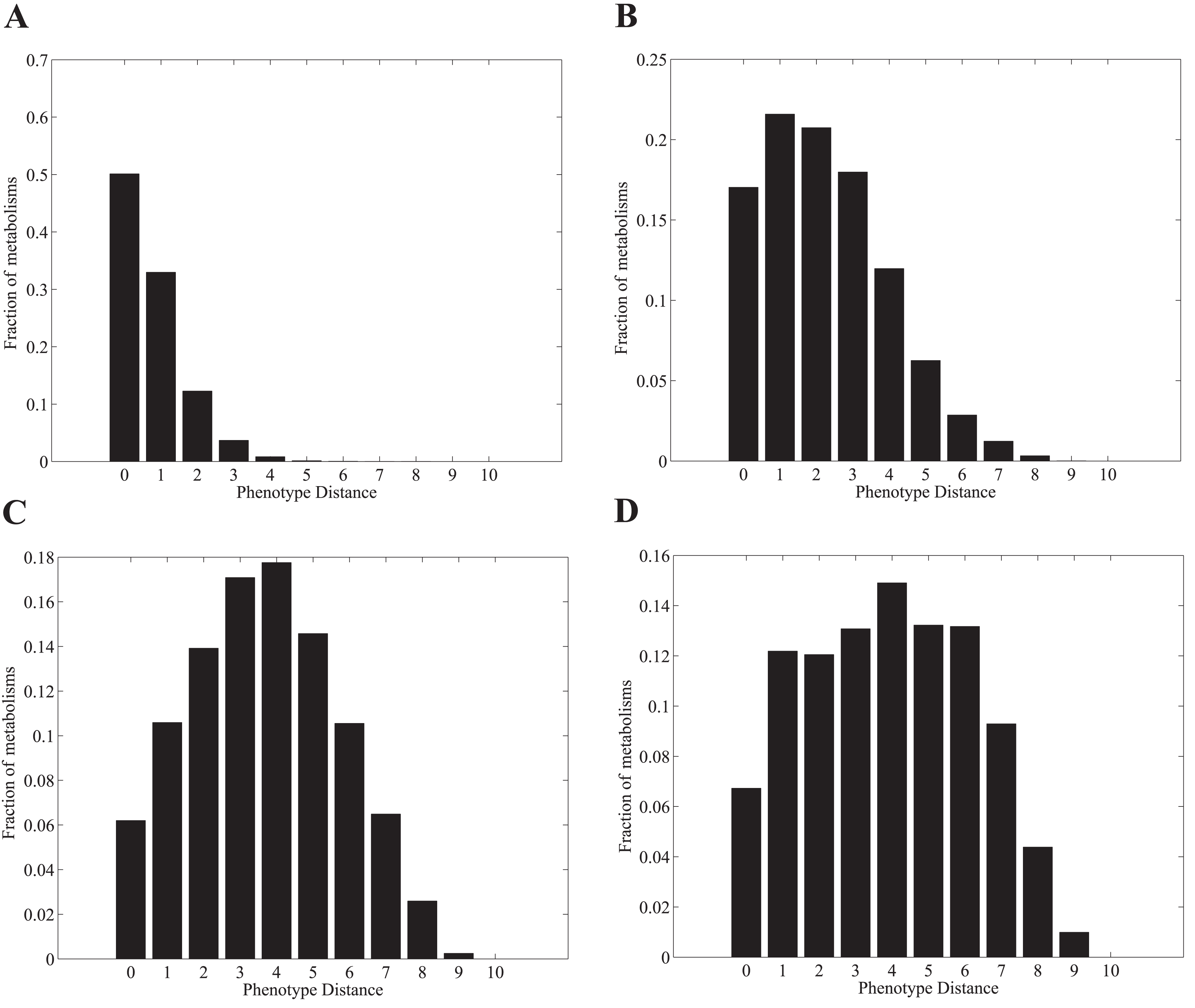

Supplement: Additional file 10: — Metabolisms viable on pyruvate as the main carbon source C can differ greatly in their viability on other carbon sources (considering only metabolisms without disconnected reactions). The figure shows a histogram of the phenotype distance (x-axis), for metabolisms without disconnected reactions with size (A) 30, (B) 35, (C) 40, and (D) 45, viable on pyruvate as carbon source C. (TIF 428 kb) [file 12918_2016_343_MOESM10_ESM.tif]

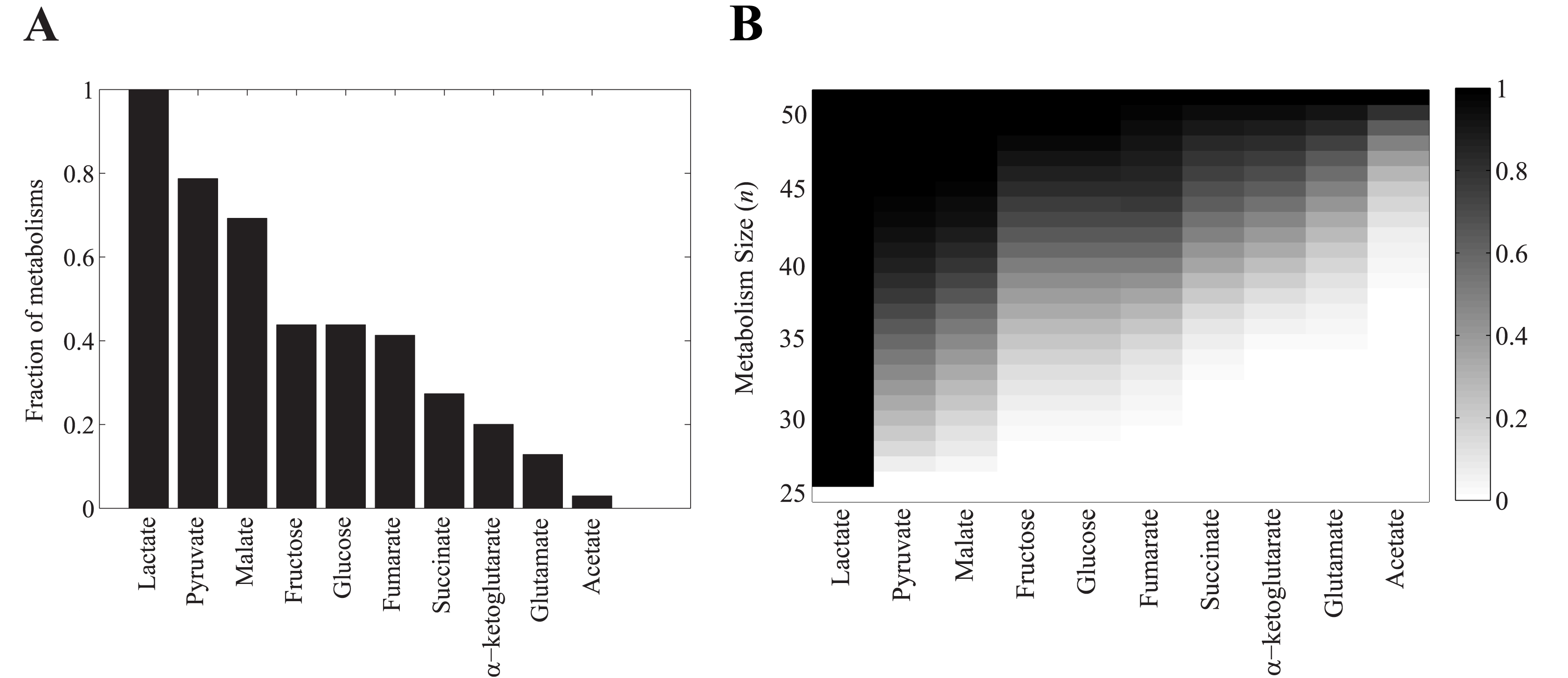

Supplement: Additional file 11: — Metabolisms viable on lactate differ in their propensity for preadaptation to other carbon sources C new. (A) The histogram shows the fraction of metabolisms viable on lactate as carbon source C that are also viable on each of the nine other carbon sources C new (x-axis). (B) As in (A), but broken down by metabolism size, and fractions of viable metabolisms are coded by shade of grey, see legend. (TIF 218 kb) [file 12918_2016_343_MOESM11_ESM.tif]

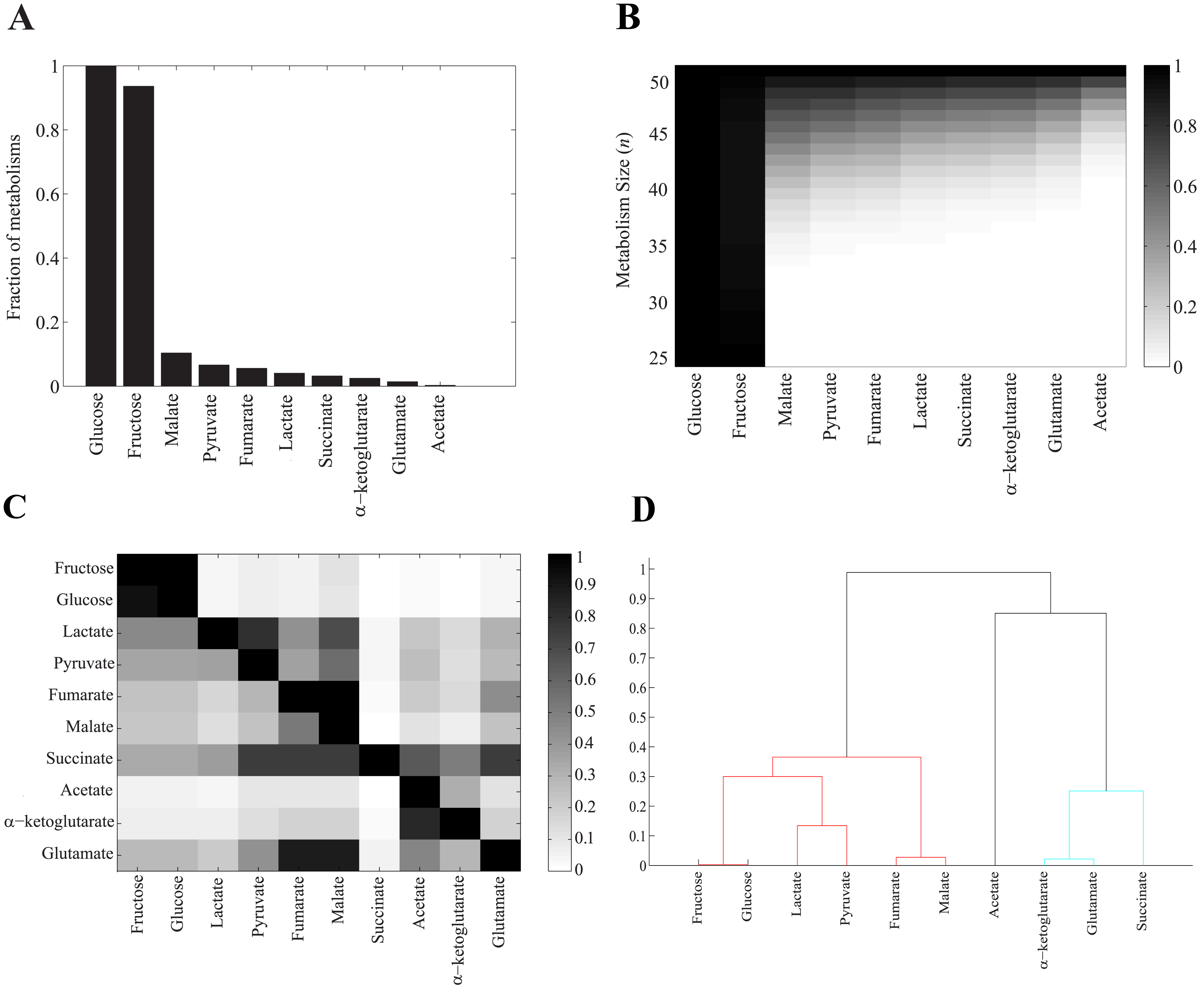

Supplement: Additional file 12: — Potential for preadaptation depends on biochemical similarity between carbon sources (considering only metabolisms without disconnected reactions). (A) The histogram shows the fraction of metabolisms (without disconnected reactions) viable on glucose as carbon source C that are also viable on each of the nine other carbon sources C new (x-axis). (B) As in (A), but broken down by metabolism size, and fractions of viable metabolisms are coded by shade of grey, see legend (C) Fraction of metabolisms (without disconnected reactions) viable on carbon source C (x-axis), that are also viable on carbon source C new (y-axis), are coded by shade of grey, see legend. (D) Dendrogram of carbon sources clustered based on their pairwise preadaptation propensity. We used UPGMA method (unweighted pair group method with arithmetic means), for clustering carbon sources. (TIF 472 kb) [file 12918_2016_343_MOESM12_ESM.tif]

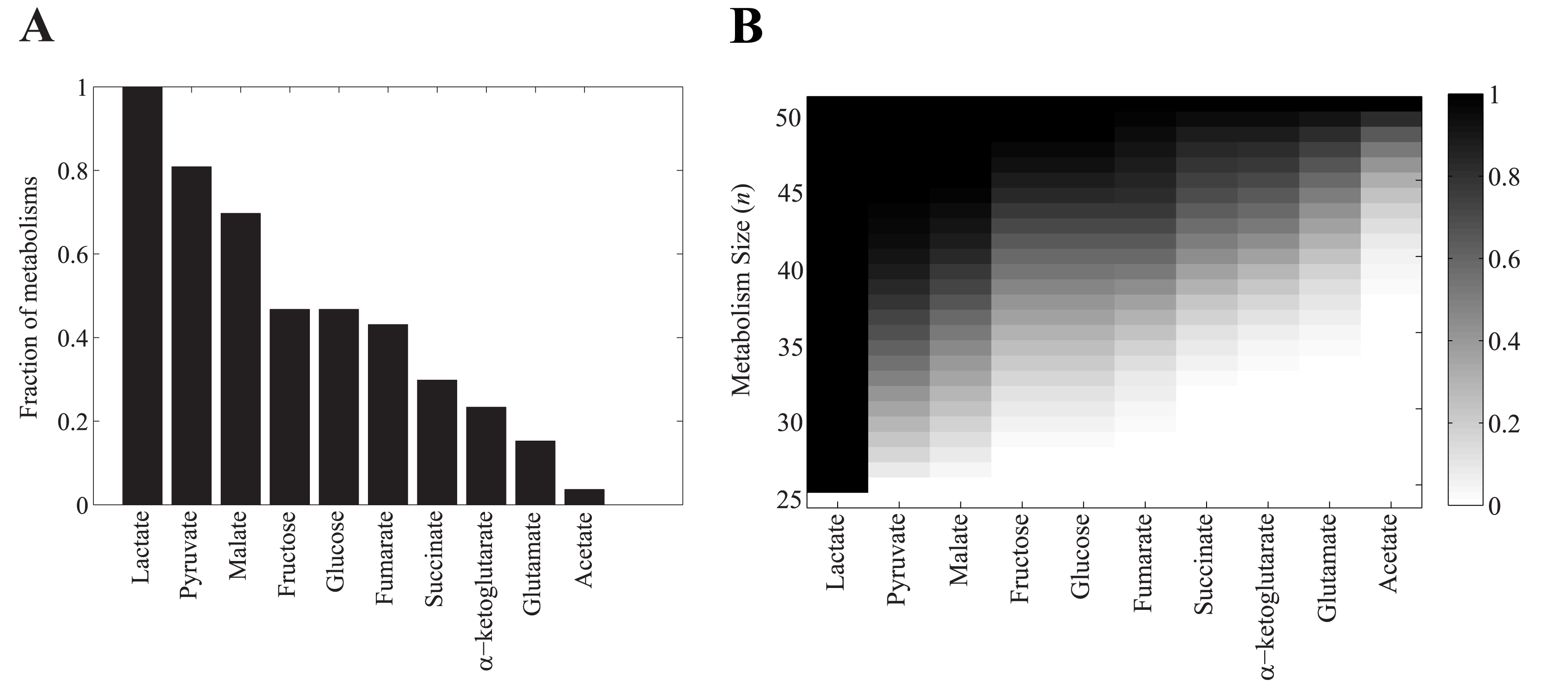

Supplement: Additional file 13: — Metabolisms viable on lactate have different potential for preadaptation to other carbon sources C new (considering only metabolisms without disconnected reactions). (A) The histogram shows the fraction of metabolisms (without disconnected reactions) viable on lactate as carbon source C that are also viable on each of the nine other carbon sources C new (x-axis). (B) As in (A), but broken down by metabolism size, and fractions of viable metabolisms are coded by shade of grey, see legend. (TIF 222 kb) [file 12918_2016_343_MOESM13_ESM.tif]

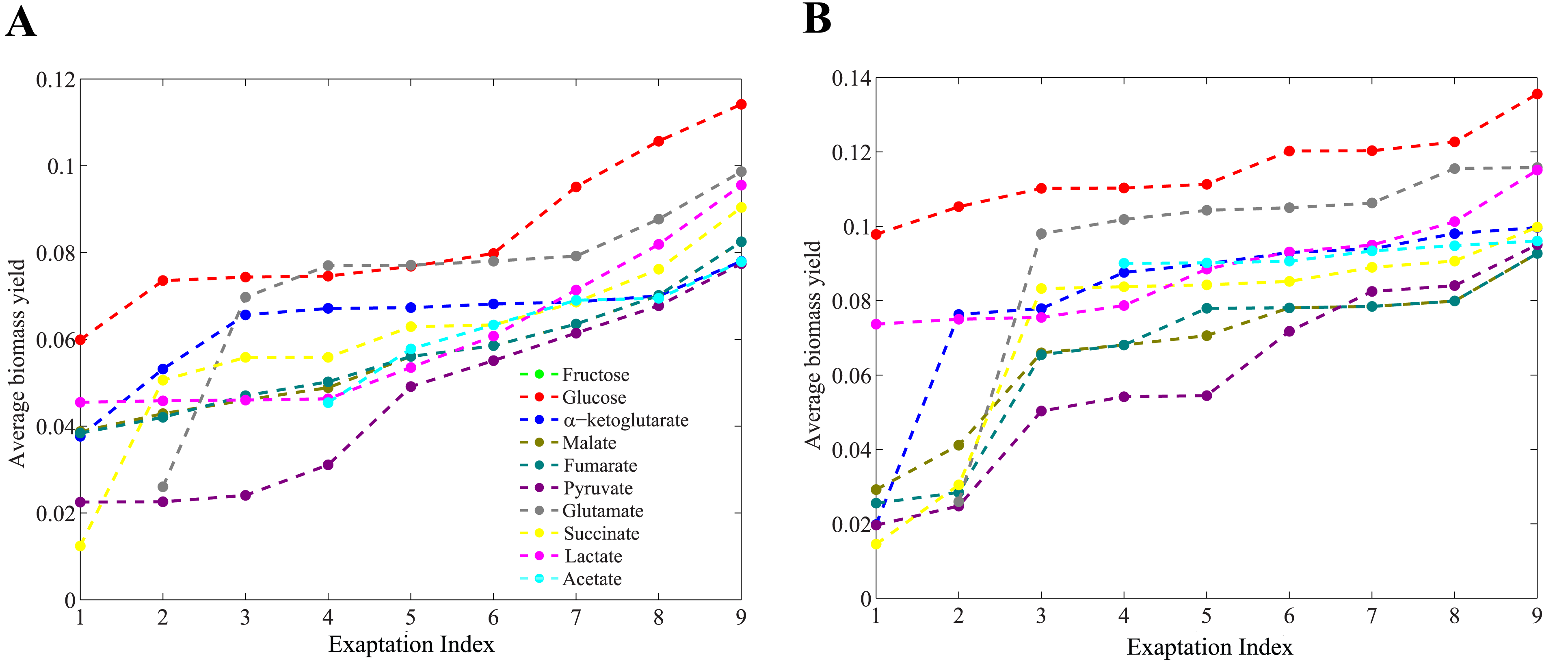

Supplement: Additional file 14: — Association between exaptation potential and biomass yield. The x-axes show the exaptation index, i.e., the number of carbon sources C new on which metabolisms viable on carbon source C (color legend) are viable. The y-axes show the average biomass yield. Data is based on metabolisms of size (A) n=35, and (B) n=45. (TIF 334 kb) [file 12918_2016_343_MOESM14_ESM.tif]

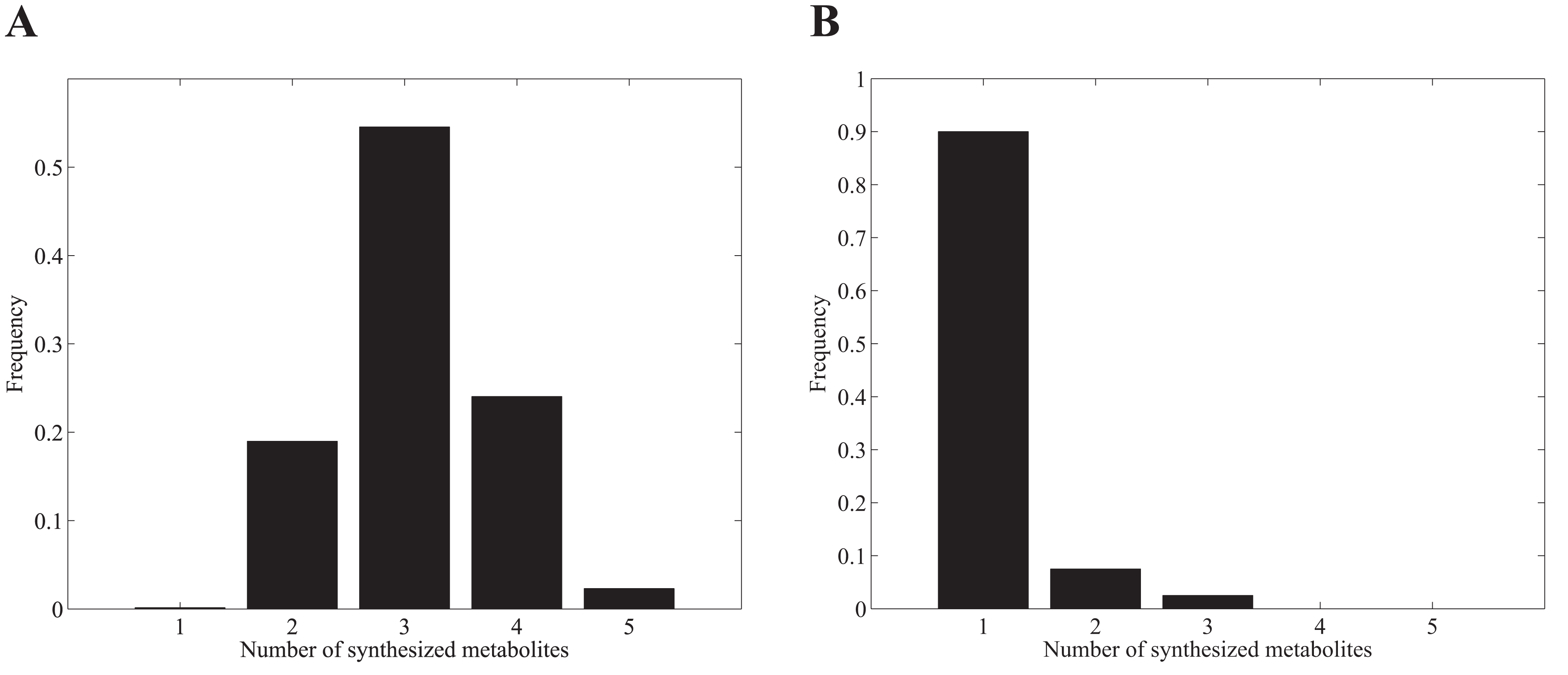

Supplement: Additional file 15: — Distribution of the number of synthesized metabolites among metabolisms viable on glucose. Fraction of metabolisms excreting a given number of metabolites (x-axis) among metabolisms viable on glucose with size (A) n=35, and (B) n=45. (TIF 174 kb) [file 12918_2016_343_MOESM15_ESM.tif]

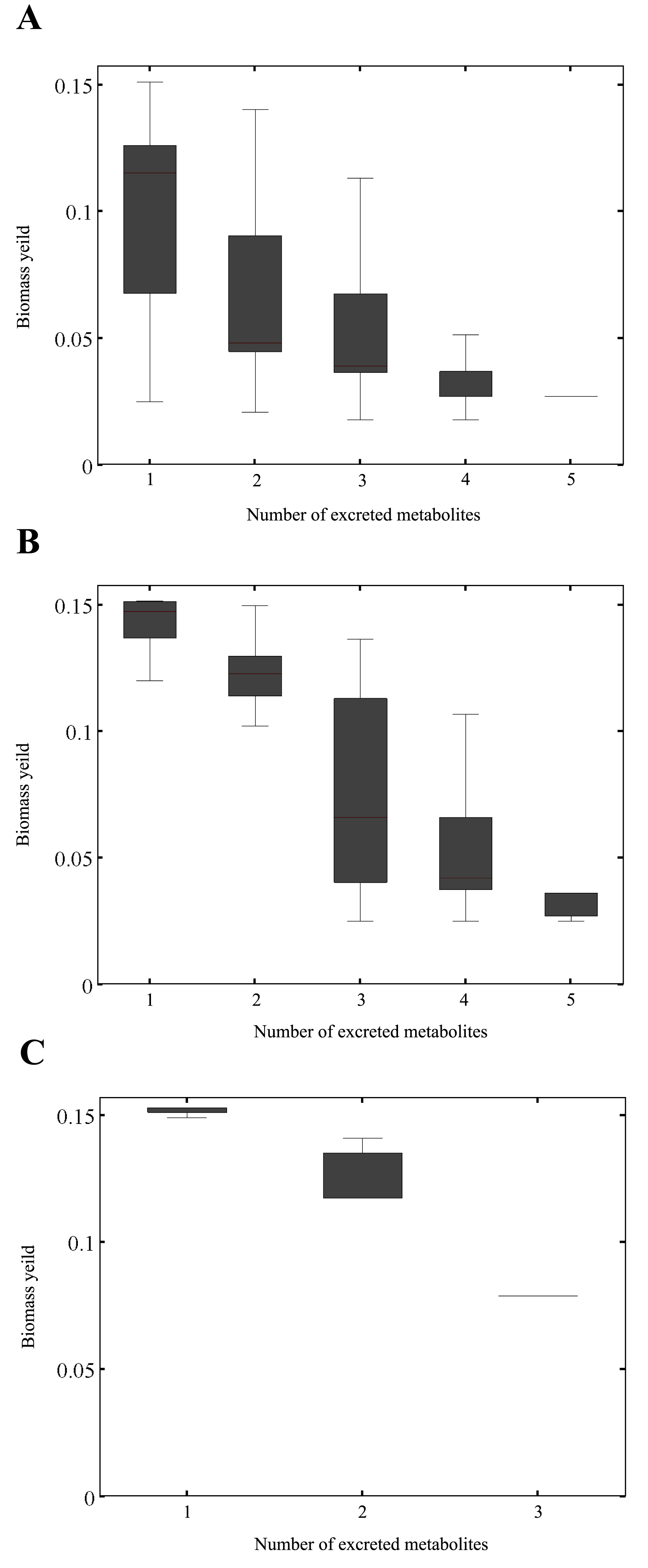

Supplement: Additional file 16: — Association between biomass yield and the number of excreted waste metabolites. The vertical axis shows the biomass yield of metabolisms of size (A) n=30, (B) n=40, and (C) n=50 viable on glucose that excrete a given number of metabolites (x-axis). Boxes span the 25-th to 75-th percentile, and whiskers indicate the maximum and minimum values. (TIF 207 kb) [file 12918_2016_343_MOESM16_ESM.tif]

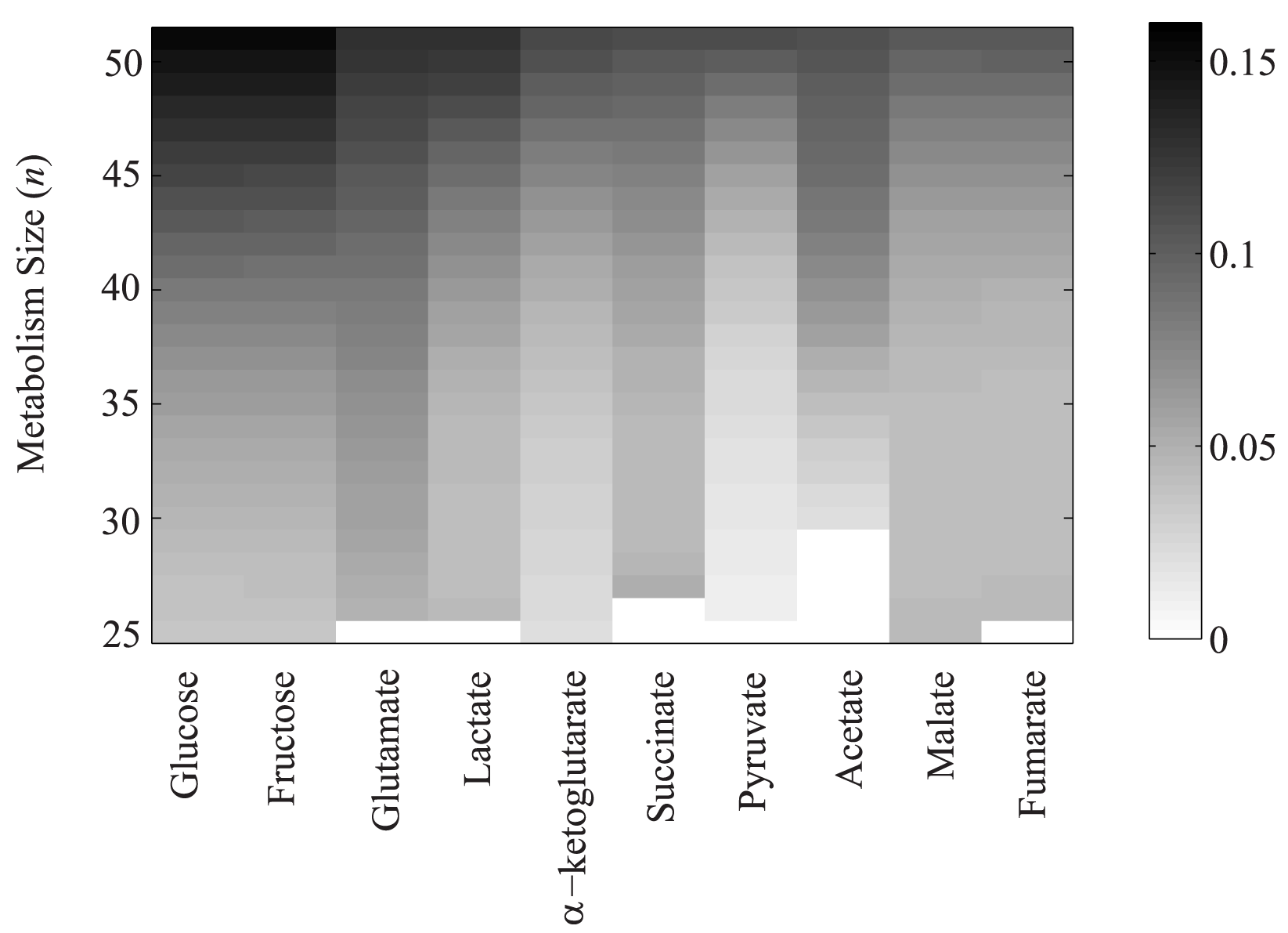

Supplement: Additional file 17: — Larger metabolisms have higher biomass yield. Mean of biomass yield among metabolisms of a given size (y-axis), that are viable on a given carbon source (x-axis), coded by shade of grey, see legend. White colors correspond to metabolisms whose size is too small for viability on C. (TIF 116 kb) [file 12918_2016_343_MOESM17_ESM.tif]
